# The SHFV nsp2 and nucleocapsid proteins recruit G3BP1 to sites of viral replication, but stress granules are not induced by the infection

**DOI:** 10.1128/jvi.00794-25

**Published:** 2025-06-23

**Authors:** Ayisha A. Lavender, Oreoluwa Solanke, Hsin-Yao Tang, Hannah E. Peck, Daryll Vanover, Philip J. Santangelo, Margo A. Brinton

**Affiliations:** 1 Department of Biology, Georgia State University 1373, Atlanta, Georgia, USA; 2 Ellen and Ronald Caplan Cancer Center, The Wistar Institute 36586, Philadelphia, Pennsylvania, USA; 3 Wallace H. Coulter Department of Biomedical Engineering, Georgia Tech and Emory University School of Medicine 189277, Atlanta, Georgia, USA; St. Jude Children's Research Hospital, Memphis, Tennessee, USA

**Keywords:** simian hemorrhagic fever virus, arterivirus, stress granules, nsp2, nucleocapsid protein, G3BP, eIF2α

## Abstract

Stress granules (SGs) are dynamic, cytoplasmic foci that form in response to environmental stresses, including viral infections, and function to restore cellular homeostasis by regulating mRNA translation, storage, and decay. To inhibit SG formation and subvert their antiviral effects, viruses from diverse families sequester or cleave G3BP1, the key SG nucleating protein. We found that an infection with simian hemorrhagic fever virus (SHFV), a member of the family *Arteriviridae*, does not induce the formation of *bona fide* SGs despite inducing phosphorylation of PKR and eIF2α. The SG proteins, G3BP1, G3BP2, TIA-1, Caprin-1, and USP10, but not the translation initiation proteins, eIF3A, eIF4G, and small ribosomal protein S6 (rpS6), were redistributed into foci located in the same intracellular region as the viral dsRNA foci. However, SGs could be induced in infected cells by exogenous inducers. LC-MS/MS analysis of the proteins co-immunoprecipitating with endogenous G3BP1 from SHFV-infected cell lysates detected multiple viral replication/transcription complex proteins. Interaction between G3BP1 and the nsp2 and N proteins of SHFV was observed in reciprocal co-immunoprecipitation assays, and colocalization was detected by IFA. A conserved FGAP motif in nsp2 and a FAEP motif in the N protein were shown to be required for interaction with G3BP1. We also detected G3BP cleavage products in the SHFV-infected cell lysates and hypothesize that cleavage is mediated by a viral protease. These findings suggest that SG formation is not induced by an SHFV infection due to recruitment of G3BP to sites of viral replication and cleavage of G3BP by viral proteins.

**IMPORTANCE:**

Eukaryotic cells shut down translation by assembling stress granules (SGs) in response to environmental stresses, including viral infections. Viruses require cellular translation machinery for protein synthesis and have developed mechanisms to subvert SG assembly. Simian hemorrhagic fever virus (SHFV), a simian arterivirus, causes asymptomatic infections in African cercopithecoid monkeys but fatal hemorrhagic fever disease in Asian macaques. Even though intracellular production of SHFV RNA activates the stress sensor, PKR, SGs are not induced. G3BP1, the main nucleating protein of SGs, is recruited to foci located near viral replication complexes through interaction with the viral proteins nsp2 and N. An FGAP motif in nsp2 and an FAEP motif in the N protein are required for interaction with G3BP1. Cleavage of G3BP1 was identified as an additional mechanism of viral counteraction of SG formation.

## INTRODUCTION

Simian hemorrhagic fever virus (SHFV) is a member of the family *Arteriviridae* and subfamily *Simarterivirinae* in the order *Nidovirales*, which also includes the family *Coronaviridae* (1). The most characterized species of the family are equine arteritis virus (EAV), lactate dehydrogenase-elevating virus (LDV), porcine reproductive and respiratory virus-1 (PRRSV-1) and PRRSV-2 and SHFV (2). SHFV was first isolated in 1964 as the etiological agent of lethal SHFV outbreaks among captive Asian macaque colonies in the United States (3). An SHFV infection in Asian macaques triggers acute hemorrhagic fever disease that is ~100% fatal within 7–14 days (4). However, infected African cercopithecoid monkeys, such as baboons, develop an asymptomatic, acute, or persistent infection (5).

SHFV is an enveloped virus, with a positive-sense, single-stranded RNA genome of 15.7 kb. The 5′ capped and 3′ polyadenylated genome encodes 16 non-structural proteins (nsps) which assemble into the membrane-associated replication/transcription complex (RTC) and 12 structural proteins, including an additional set of minor structural proteins (GP 2a′, E′, GP 3′, and GP 4′) present only in simian arterivirus genomes (6). The nsps are encoded in the 5′ open reading frame 1a (ORF 1a) and ORF 1b and are translated into polyprotein 1a (pp1a) and pp1ab, with the latter resulting from a ribosomal frame shift at the 3′ end of ORF 1a (2). The nsps are auto-proteolytically processed by four active papain-like proteases (PLPs) found in nsp1α, nsp1β, nsp1γ, and nsp2, and the chymotrypsin-like serine protease (SP) located in nsp4 (7–10). In addition to its function as a protease, nsp2, along with nsp3 and nsp5, modifies the endoplasmic reticulum (ER) membranes to form double-membrane vesicles, in which the RTC facilitates viral RNA replication and transcription (11–13). The structural proteins, located in the 3′ third of the genome, are expressed from a 5′−3′ coterminal, nested set of subgenomic mRNAs that are generated by discontinuous minus-strand transcription from overlapping ORFs (14).

The cellular stress-related kinase, protein kinase R (PKR), is activated by cytosolic double-stranded (ds) RNA including base-paired regions in single-stranded viral genomic RNAs (15). Once activated, PKR phosphorylates the α subunit of the eukaryotic translation initiation factor 2 (eIF2), which reduces the availability of eIF2-GTP-Met-tRNA ternary complexes leading to inhibition of global cap-dependent translation initiation and triggering the assembly of stress granules (SGs) (16). eIF2α can also be phosphorylated by four additional kinases, protein kinase R (PKR)-like ER kinase (PERK), general control non-derepressible-2 (GCN2), heme-regulated inhibitor (HRI), and microtubule affinity regulating kinase 2 (MARK2) (17), in response to other environmental stresses. SGs are discrete, highly dynamic, cytoplasmic foci that form in response to environmental stresses and function to restore cellular homeostasis by regulating mRNA translation, storage, and decay (18, 19). SGs are comprised of non-polysomal mRNA bound to the 40S ribosomal subunit and translation initiation factors, such as eIF3A, eIF4G, eIF4A, eIF4E, and PABP. SGs also contain multiple RNA-binding proteins (RBPs) and other types of proteins that vary with cell type and the duration and type of stress (20). Ras GTPase-activating protein binding protein 1 (G3BP1), G3BP2, T-cell internal antigen 1 (TIA-1), and T-cell internal antigen-related protein (TIAR) are core RBPs present in *bona fide* SGs due to their function in SG nucleation (21, 22). Additional RBPs, such as cell cycle-associated protein 1 (Caprin-1) and ubiquitin-specific peptidase 10 (USP10), are also commonly found in SGs and are known to competitively bind G3BP1 to promote or inhibit SG assembly, respectively (23). SGs have been reported to play an antiviral role in cells infected with many types of viruses. SGs shut down cellular host translation machinery, which is needed for viral protein synthesis. Although a few viruses are able to replicate in the presence of SGs (24, 25), many viruses from diverse families have evolved strategies to inhibit or remodel SG formation to promote viral replication. G3BP1, the primary nucleating SG protein, is often targeted for sequestration by viral proteins or is cleaved by viral proteases to inhibit SG formation (26).

PRRSV-1 infections were previously shown to induce *bona fide* SGs through activation of the PERK/eIF2α pathway (27), but the induction of SGs by infections with other arteriviruses, including SHFV, has not yet been explored. In the present study, we showed that an SHFV infection in MA104 cells induced PKR and eIF2α phosphorylation, but *bona fide* SG foci were not detected at any time after infection. While some SG RBPs, such as G3BP1, G3BP2, TIA-1, Caprin-1, and USP10, were redistributed into foci located in the same intracellular region as the viral dsRNA foci, the translation initiation proteins, eIF3A, eIF4G, and small ribosomal protein S6 (rpS6), were not detected in these foci. SGs could be induced in SHFV-infected cells by treatment with sodium arsenite (SA) or dithiothreitol (DTT), but the number was significantly reduced compared to uninfected treated cells. LC-MS/MS data indicated that SHFV nsps were the most highly enriched proteins co-precipitating with G3BP1 from SHFV-infected cell lysates. Reciprocal co-immunoprecipitation assays and IFAs confirmed an interaction between G3BP1 and nsp2 and showed that a conserved FGAP motif present in the nsp2 proteins of most of the simian arteriviruses was required for this interaction. We also showed that G3BP1 interacts with the SHFV nucleocapsid (N) protein via a different motif, FAEP, found only in the N proteins of the simian arteriviruses. These data indicate that an SHFV infection prevents the assembly of *bona fide* SGs by recruiting G3BP1 to areas with viral replication complexes through interaction with the viral nsp2 and N protein. We also observed the accumulation of a G3BP1 and a G3BP2 cleavage product in SHFV-infected cells, suggesting an additional viral strategy for hindering SG formation.

## RESULTS

### An SHFV infection induces PKR and eIF2-α phosphorylation

**Fig 1:**
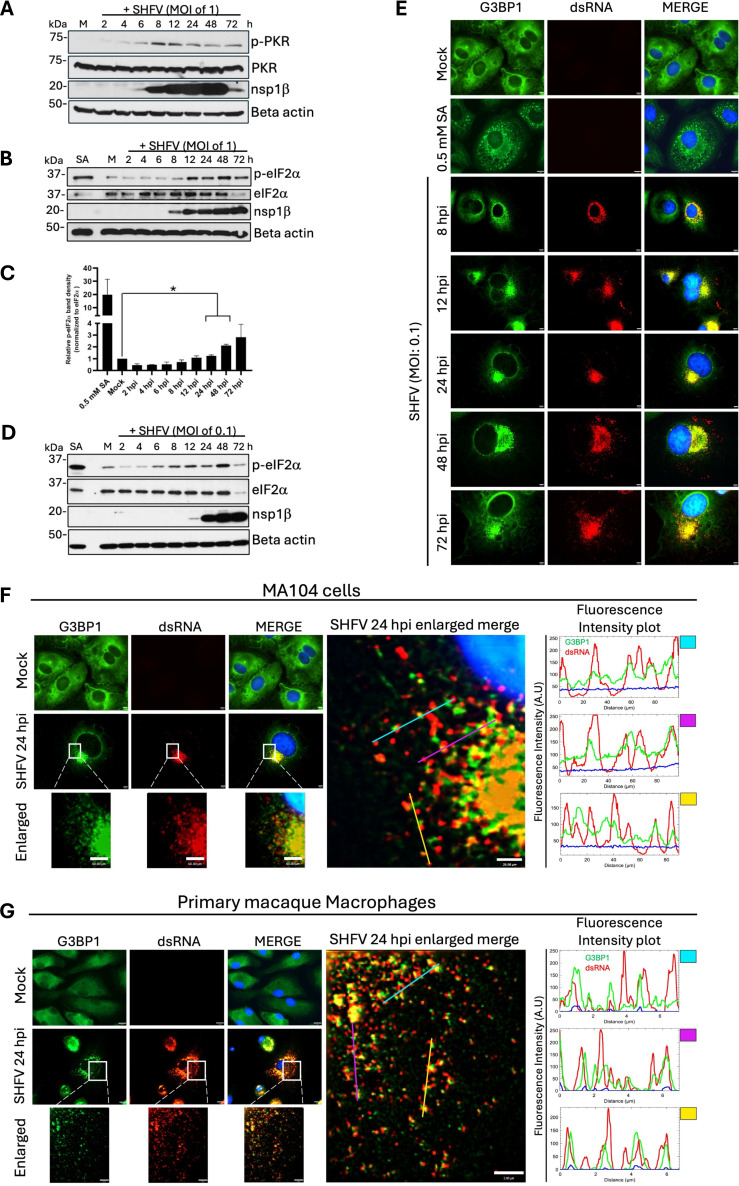
An SFHV infection induces activation of the SG response pathway and redistribution of G3BP1 in SHFV-infected cells. (**A–D**) MA104 cells were mock-infected or infected with SHFV at the indicated MOI. Uninfected cells were treated with SA (0.5 mM) for 30 min at 37°C. Whole-cell lysates were harvested in 1× RIPA buffer at the indicated times and used for WB analysis with antibodies specific for (**A**) PKR, p-PKR, SHFV nsp1β or (**B, D**) eIF2α, p-eIF2α, or SHFV nsp1β. (**C**) Relative intensity of p-eIF2α protein bands in mock-infected, SA-treated or SHFV-infected cells. WB band intensity was quantified using ImageJ software and normalized to total eIF2α. Values are ±SEM and significance was analyzed using an unpaired Student’s *t*-test; *P ≤ 0.05. The data shown are representative of three independent experiments. (**E**) MA104 cells were mock-infected or infected with SHFV (MOI of 0.1). Uninfected MA104 cells were treated with 0.5 mM of SA for 30 min at 37°C. At the indicated times, cells were fixed, permeabilized, and incubated with anti-G3BP1 antibody (green) and anti-dsRNA antibody (red). Nuclei were stained with Hoechst 33342 (blue). (**F**) Mock-infected images and enlarged portions of the SHFV 24 hpi images shown in panel **E**. Fluorescence intensity profiles of the G3BP1 (green) and dsRNA (red) fluorescent signals along three different colored cross-section lines in the enlarged merged image of infected MA104 cells were generated with ImageJ software. (**G**) Primary macaque MΦs were mock-infected or infected with SHFV (MOI of 0.1). The cells were fixed and processed for IFA at 24 hpi. Fluorescence intensity profiles of the G3BP1 (green) and dsRNA (red) fluorescent signals along three different colored cross-section lines of an enlarged merged IFA image are shown. In this and subsequent figures, images were obtained with a Zeiss Axio Observer Z1 fluorescent microscope and a 40× objective. Scale bars, 100 µm. Enlarged images scale bars, 50 µm.

We previously reported that approximately 30–50% of the total RNA found in SHFV-infected MA104 cells at 8 and 18 h post infection (hpi) and primary macaque macrophages (MΦ) at 7 and 16 hpi is of viral origin (28). Since the eIF2α kinase, PKR, is activated by viral dsRNA, we investigated the phosphorylation levels of PKR and eIF2α in SHFV-infected MA104 cells (MOI of 1). Cell lysates were harvested at different times between 2 and 72 hpi. As a positive control, uninfected cells were treated with SA, an oxidative stressor, to induce eIF2α phosphorylation. Western blot (WB) analysis detected low levels of PKR phosphorylation by 4 hpi, with the highest levels detected between 8 and 12 hpi (Fig. 1A). Phosphorylated eIF2α levels increased above background levels by 24 hpi and remained high through 48 hpi (Fig. 1B and C). A similar increase in phosphorylated eIF2α levels was observed in MA104 cells infected at an MOI of 0.1 (Fig. 1D). These data indicate that the production of viral RNA in SHFV-infected cells activates PKR and leads to the phosphorylation of eIF2α.

### G3BP1 localizes to the sites of viral replication in SHFV-infected cells

An infection with the arterivirus PRRSV was previously shown to induce SG formation in MARC-145 cells in a PERK-dependent manner (27). To determine whether an SHFV infection induced the formation of SGs via PKR activation, MA104 cells and primary macaque MΦs were infected with SHFV at an MOI of 0.1, and at various times post-infection, the intracellular distribution of G3BP1 was examined by an immunofluorescence assay (IFA). The SG-nucleating protein, G3BP1, is present in the core and shell of SGs and is a widely accepted marker for SGs (29). Uninfected cells served as a negative control, and uninfected cells treated with SA were used as a positive SG control. Viral replication complexes were identified with anti-dsRNA antibody. As expected, distinct G3BP1 foci, characteristic of SGs, were observed throughout the cytoplasm of SA-treated cells, whereas a diffuse cytoplasmic distribution of G3BP1 was observed in the uninfected, untreated cells. In the cytoplasm of SHFV-infected cells, G3BP1 localized to the same regions as the viral dsRNA throughout the course of the infection (Fig. 1E). The viral dsRNA and G3BP1 were initially distributed symmetrically in the perinuclear region, but by 12 hpi, dsRNA and G3BP1 were asymmetrically distributed in the majority of the infected cells (Fig. 1E). Enlarged images showed that G3BP1 and dsRNA were primarily located in separate foci, although complete colocalization of the two types of foci was occasionally observed (Fig. 1F). A similar redistribution pattern for G3BP1 was detected in SHFV-infected primary macaque MΦs (Fig. 1G). Fluorescence intensity plots across three different regions (indicated by the colored solid lines in the enlarged merge images) showed either adjacent peaks of individual G3BP1 or dsRNA foci or overlapping peaks indicating colocalization of these foci (Fig. 1F and G, right panels).

### An SHFV infection does not induce SGs in MA104 cells

**Fig 2:**
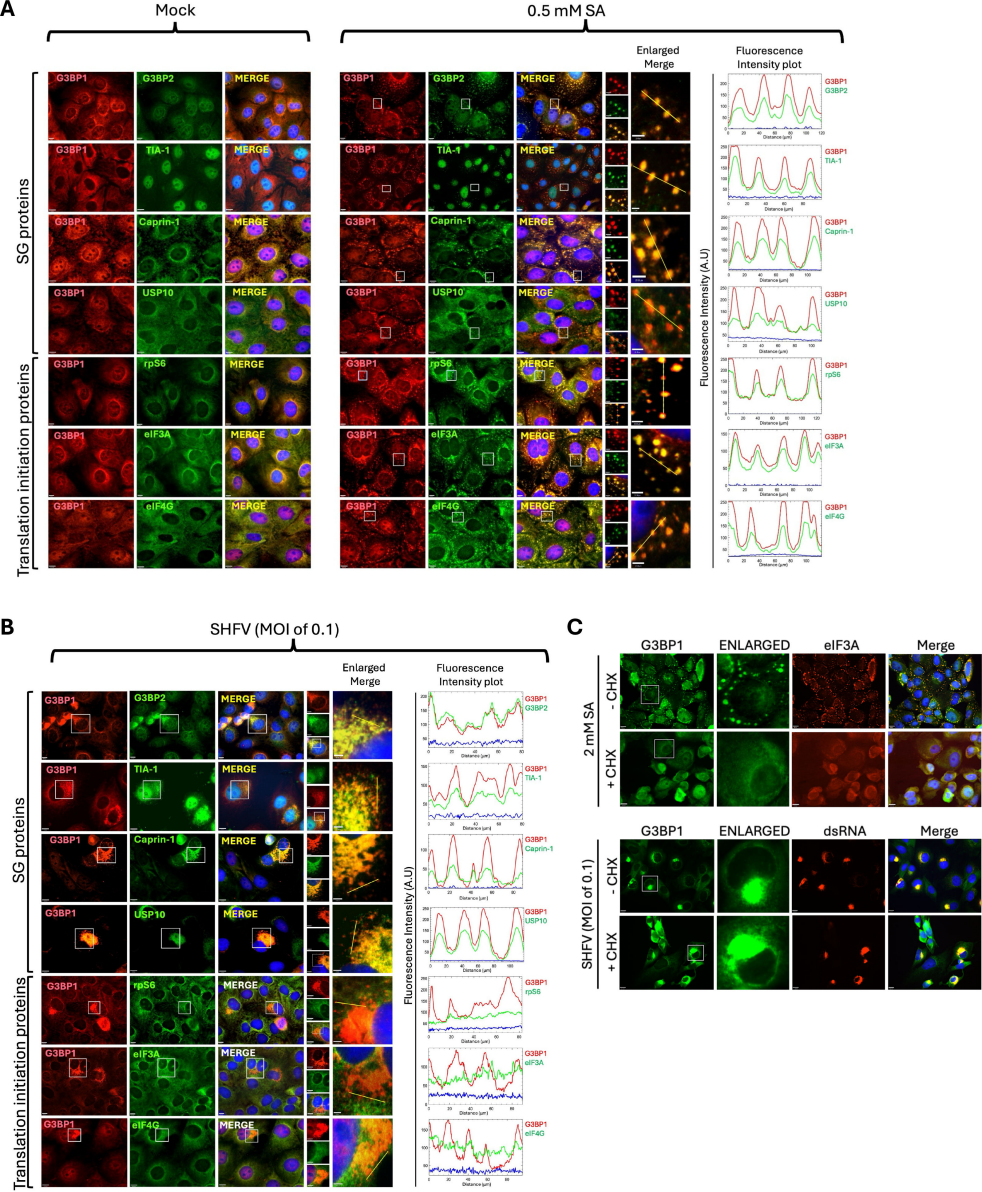
G3BP1 foci in SHFV-infected cells are not *bona fide* SGs. (**A**) MA104 cells were mock-infected and 28 h later, the cells were incubated with SA (0.5 mM) for 30 min. (**B**) MA104 cells were infected with SHFV (MOI of 0.1) and at 28 hpi, the cells were incubated with SA (0.5 mM) for 30 min. Cells were fixed, permeabilized, and incubated with antibodies specific for G3BP1 (red) and for either G3BP2, TIA-1, Caprin-1, USP10, rpS6, eIF3A, or eIF4G (green). Nuclei were stained with Hoechst 33342 (blue). Fluorescence intensity profiles of G3BP1 (red) and either G3BP2, TIA-1, Caprin-1, USP10, rpS6, eIF3A, or eIF4G signals (green) along the yellow cross-section lines in the enlarged merge images of uninfected SA-treated (**A**) and SHFV-infected (**B**) MA104 cells. (**C**) MA104 cells were either mock-infected (top panel) or infected with SHFV (MOI of 0.1) for 24 h (bottom panel). At 24 h, uninfected cells were treated with 2 mM SA for 45 min. Replicate uninfected cells were treated with 2 mM SA for 15 min. The uninfected, SA-treated (15 min) and SHFV-infected cells were incubated with CHX (100 µg/mL) for 1 h. Cells were fixed, permeabilized, and incubated with antibodies specific for G3BP1 (green) and eIF3A (red) or dsRNA (red). Nuclei were stained with Hoechst 33342 (blue).

To determine whether the redistributed G3BP1 foci in the SHFV-infected cells are in fact *bona fide* SGs, MA104 cells were infected with SHFV at an MOI of 0.1 for 28 h, and the colocalization of additional SG proteins with G3BP1 was analyzed by IFA. Uninfected cells were treated with 0.5 mM SA as a positive control to confirm colocalization of each SG protein with G3BP1. All 7 of the investigated proteins colocalized with G3BP1 in SG foci in the SA-treated cells (Fig. 2A). In contrast, the SG proteins, G3BP2, TIA-1, Caprin-1, and USP10 colocalized with the redistributed G3BP1 in the infected cells, while the translation pre-initiation proteins, eIF4G and eIF3A, and the small ribosomal protein, rpS6, remained diffusely dispersed in the cytoplasm, indicating that stalled non-polysomal mRNAs were not associated with the G3BP1 foci in the infected cells (Fig. 2B).

Cycloheximide (CHX) is a translation elongation inhibitor that is used as a tool to identify *bona fide* SGs, as it traps ribosomes on mRNA transcripts and limits the availability of non-polysomal mRNA that can assemble into SGs. Preformed SGs dissolve due to their dynamic nature, and new SGs are inhibited from assembling in the presence of CHX (21). To further characterize the G3BP1 foci in SHFV-infected cells, MA104 cells infected with SHFV for 24 h were incubated with CHX (100 µg/mL) for 1 h and then processed for IFA to detect the intracellular distribution of G3BP1. Uninfected cells treated with SA (2 mM) for 45 min to induce SGs and replicate uninfected cells that were first treated with SA for 15 min, and then incubated with CHX (100 µg/mL) for 1 h were used as controls. The preformed SGs induced by SA in uninfected cells were not detected following the addition of CHX (Fig. 2C, top panels). However, the asymmetrically redistributed G3BP1 foci in the SHFV-infected cells were still detected following CHX treatment, indicating that the characteristics of SHFV-induced G3BP1 foci were not consistent with those of dynamic *bona fide* SGs (Fig. 2C, bottom panel). These data provide evidence confirming that an SHFV infection does not induce the formation of *bona fide* SGs.

**Fig 3:**
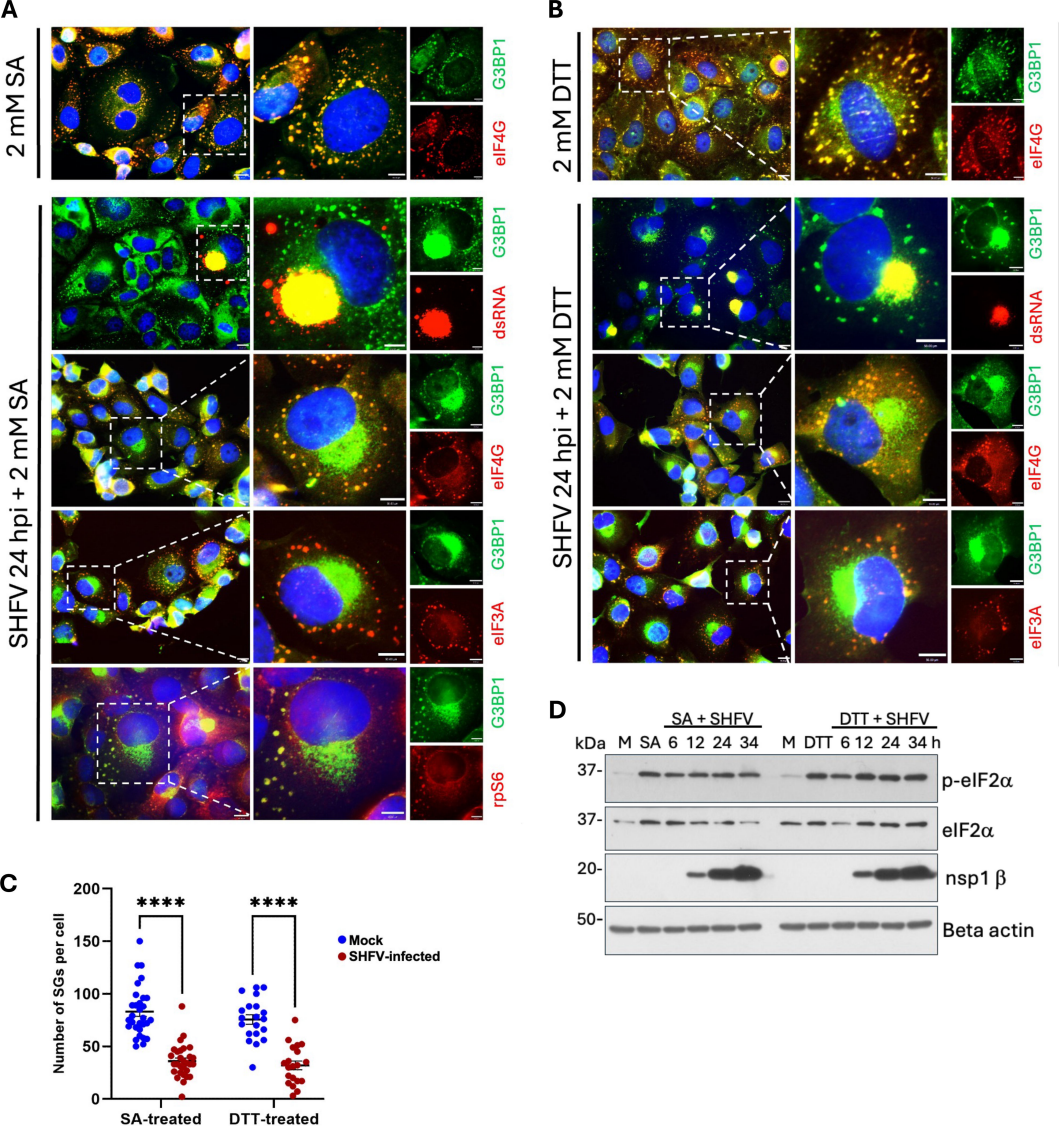
The number of SGs induced by SA or DTT was reduced by an SHFV infection. MA104 cells were mock-infected or infected with SHFV (MOI of 0.1). At 24 h, mock-infected cells were incubated with (**A**) SA (2 mM) or (**B**) DTT (2 mM) for 45 min. Cells were fixed, permeabilized, and incubated with antibodies specific for G3BP1 (green) and for either dsRNA, eIF4G, eIF3A, or rpS6 (red). Nuclei were stained with Hoechst 33342 (blue). (**C**) A minimum of 30 (SA-treated) or 20 (DTT-treated) cells was used for quantification of SGs in either mock-infected or SHFV-infected cells. Quantification of SGs in individual cells was performed by manual counting of foci that were positive for G3BP1 and either eIF4G, eIF3A, or rpS6. Values shown are ±SEM from three independent experiments, and significance was analyzed using a two-way ANOVA test; ****P ≤ 0.0001. (**D**) MA104 cells were mock-infected or infected with SHFV (MOI of 0.1). At different times after infection, mock-infected and infected cells were incubated with either SA (2 mM) or DTT (2 mM) for 45 min and whole-cell lysates were harvested and analyzed by WB using antibodies specific for p-eIF2α, eIF2α, or SHFV nsp1β.

To determine whether an SHFV infection also can inhibit SG formation by exogenous SG inducers, MA104 cells were infected with SHFV at an MOI of 0.1 for 24 h and then incubated with SA (2 mM) or DTT (2 mM) for 45 min before being processed for IFA. The translation pre-initiation proteins, eIF4G and eIF3A, and the small ribosomal protein, rpS6, colocalized with G3BP1 in distinct SGs scattered in the cytoplasm of infected cells treated with SA or DTT but did not colocalize with the asymmetrically redistributed G3BP1 foci detected in the same cells (Fig. 3A and B). The average number of SGs detected in the infected SA-treated cells (36 SGs per cell) or infected DTT-treated cells (32 SGs per cell) was significantly reduced in comparison to the number in uninfected cells treated with SA (83 SGs per cell) or DTT (76 SGs per cell) (Fig. 3C). To determine whether treatment of SHFV-infected cells with an exogenous SG inducer alters the phosphorylation levels of eIF2α, MA104 cells were either mock-infected or infected with SHFV (MOI of 0.1), and at various times post-infection, cells were treated with SA (2 mM) or DTT (2 mM) for 45 min and then harvested for WB analysis. The levels of eIF2α phosphorylation in infected cells were only slightly increased by treatment with SA or DTT by 12 hpi (Fig. 3D), suggesting that an SHFV infection prevents SG formation downstream of early stress response signaling. These data indicate that an SHFV infection does not inhibit exogenous inducers from inducing the formation of *bona fide* SGs but does significantly reduce the number of SG foci that are formed.

### Multiple SHFV replication/transcription complex proteins co-precipitate with G3BP1

**Fig 4:**
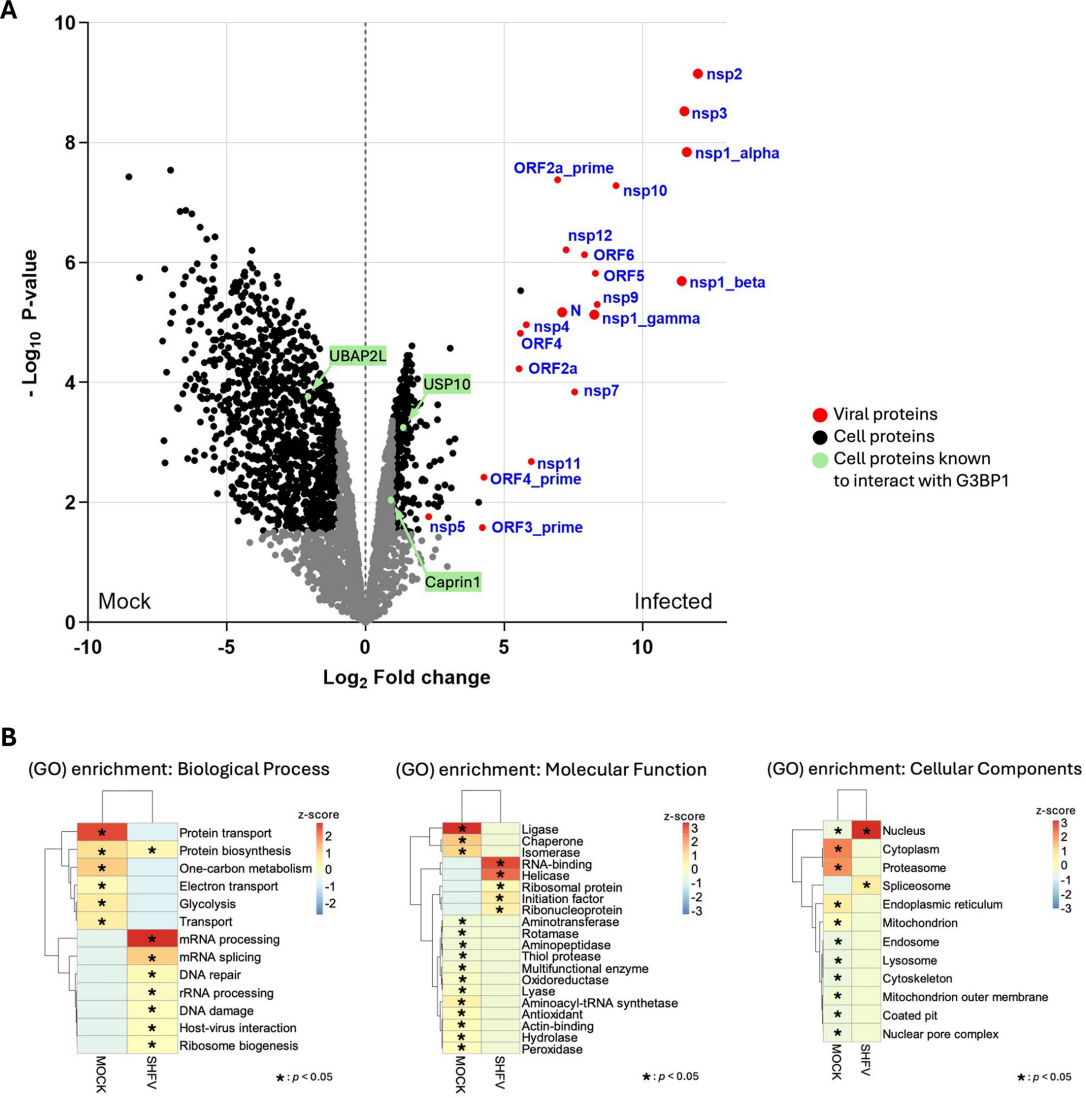
SHFV nonstructural proteins are highly enriched by G3BP1 immunoprecipitation of infected cell lysates. Lysates prepared from MA104 cells that were mock-infected or infected with SHFV (MOI of 0.1) for 24 h were immunoprecipitated with rabbit anti-G3BP1 antibody. The immunoprecipitated proteins were analyzed by LC-MS/MS. (**A**) A volcano plot showing the degree of enrichment of proteins co-precipitated with G3BP1 from mock-infected or infected MA104 cells. Black dots (cellular proteins) and red dots (viral proteins) indicate proteins with a significant enrichment indicated by a fold change (FC) ≥2 and an adjusted *P* value < 0.05 in a mock vs infected condition comparison. Gray dots indicate cellular proteins with a FC or *P* value below the set threshold. Green dots indicate cellular proteins known to interact with G3BP1. (**B**) A gene ontology (GO) enrichment analysis was performed on proteins significantly enriched in the G3BP1 precipitates from either mock-infected or SHFV-infected cells. The genes were classified into biological process (BP), molecular function (MF), and cellular component (CC) categories. *P < 0.05.

Previous studies have shown that members of different virus families utilize a variety of strategies to counteract SG assembly and their antiviral effects (30). Sequestration of G3BP1 by a viral protein has been documented for multiple alphaviruses, including Sindbis virus (SINV), Semliki Forest virus (SFV), chikungunya virus (CHIKV), and Mayaro virus (MAYV), as well as for severe acute respiratory syndrome coronavirus 2 (SARS-CoV-2) and Zika virus (ZIKV) (26, 31–33). To determine whether G3BP1 is recruited to the viral replication complexes by an interaction with a viral protein, we mock-infected or infected MA104 cells at an MOI of 0.1 for 24 h and then immunoprecipitated whole-cell lysates with anti-G3BP1 antibody followed by analysis of the precipitated proteins by LC-MS/MS. Cellular and viral proteins that were enriched by co-precipitation with G3BP1 from the mock-infected and SHFV-infected cell lysates are indicated on the volcano plot. Proteins with a fold change of 2 or greater and a *P* value less than 0.05 are indicated as black dots (cellular proteins) or red dots (viral proteins), whereas proteins not meeting these thresholds are indicated as gray dots. SHFV nsp2, nsp3, nsp1α, and nsp1β were the most significantly enriched viral proteins co-precipitating with G3BP1 in the infected cells (Fig. 4A). Additional viral nsps and structural proteins, including the nucleocapsid protein, also co-precipitated with G3BP1. In addition, fewer cellular proteins co-precipitated with G3BP1 in the infected cells compared to the number in the uninfected cells. The degree of enrichment of cellular proteins known to bind to G3BP1 and function to either promote (Caprin-1 and UBAP2L) (34) or inhibit (USP10) (23) SG formation was compared in mock-infected and SHFV-infected G3BP1 precipitates. UBAP2L was significantly enriched in the mock-infected lysate precipitates. USP10 was significantly enriched, but Caprin1 was not, in the SHFV-infected lysate precipitates (Fig. 4A; Table S1).

To investigate differences between the sets of cellular proteins co-precipitated with G3BP1 in SHFV-infected and uninfected cells, a gene ontology (GO) enrichment analysis was performed using the DAVID online database, and the results were classified into biological process (BP), molecular function (MF), and cellular component (CC) categories. The BP analysis indicated that the co-precipitated cellular proteins from the infected cells were mainly involved in mRNA processing and mRNA splicing pathways, while the cellular proteins co-precipitated from the mock-infected cells were primarily involved in protein transport and protein biosynthesis pathways. The MF analysis showed that the co-precipitated cellular proteins from the infected cell lysates were involved in RNA binding and unwinding, whereas the enriched cellular proteins that co-precipitated from the mock-infected cell lysates were ones with ligase or protein chaperoning activities. The CC analysis indicated that nuclear and spliceosome proteins were enriched among those co-precipitated from the infected cells, while cytoplasmic proteins were primarily enriched among those co-precipitated from the uninfected cells (Fig. 4B). These data suggest that the G3BP1 interactome is greatly modified by an SHFV infection.

### G3BP1 is recruited to viral replication complexes through interaction with SHFV nsp2 and the nucleocapsid protein

We next investigated interactions between G3BP1 and individual SHFV nsps using reciprocal co-immunoprecipitation assays. The PRRSV nsp1β was recently shown to co-precipitate with G3BP1 in infected MARC 145 cells (35). SHFV expresses three nsp1 proteins, nsp1α, nsp1β, and nsp1γ, and all three of these proteins were enriched in the liquid chromatography-tandem mass spectrometry (LC-MS/MS) analysis (Fig. 4A; Table S1). The possibility of an interaction of G3BP1 with each individual nsp1 protein, as well as with the most enriched viral proteins, nsp2 and nsp3, was investigated. We also investigated a potential interaction between G3BP1 and the SHFV N protein that also co-precipitated with G3BP1, because recent studies showed that the SARS-CoV-2 N protein directly binds G3BP1 and this interaction contributes to the inhibition of SG formation (31, 36). Although the N protein is not required for replication or transcription (37), it was shown to assemble into a filamentous network of tubules near the sites of viral replication in EAV-infected cells as the infection progressed (38).

**Fig 5:**
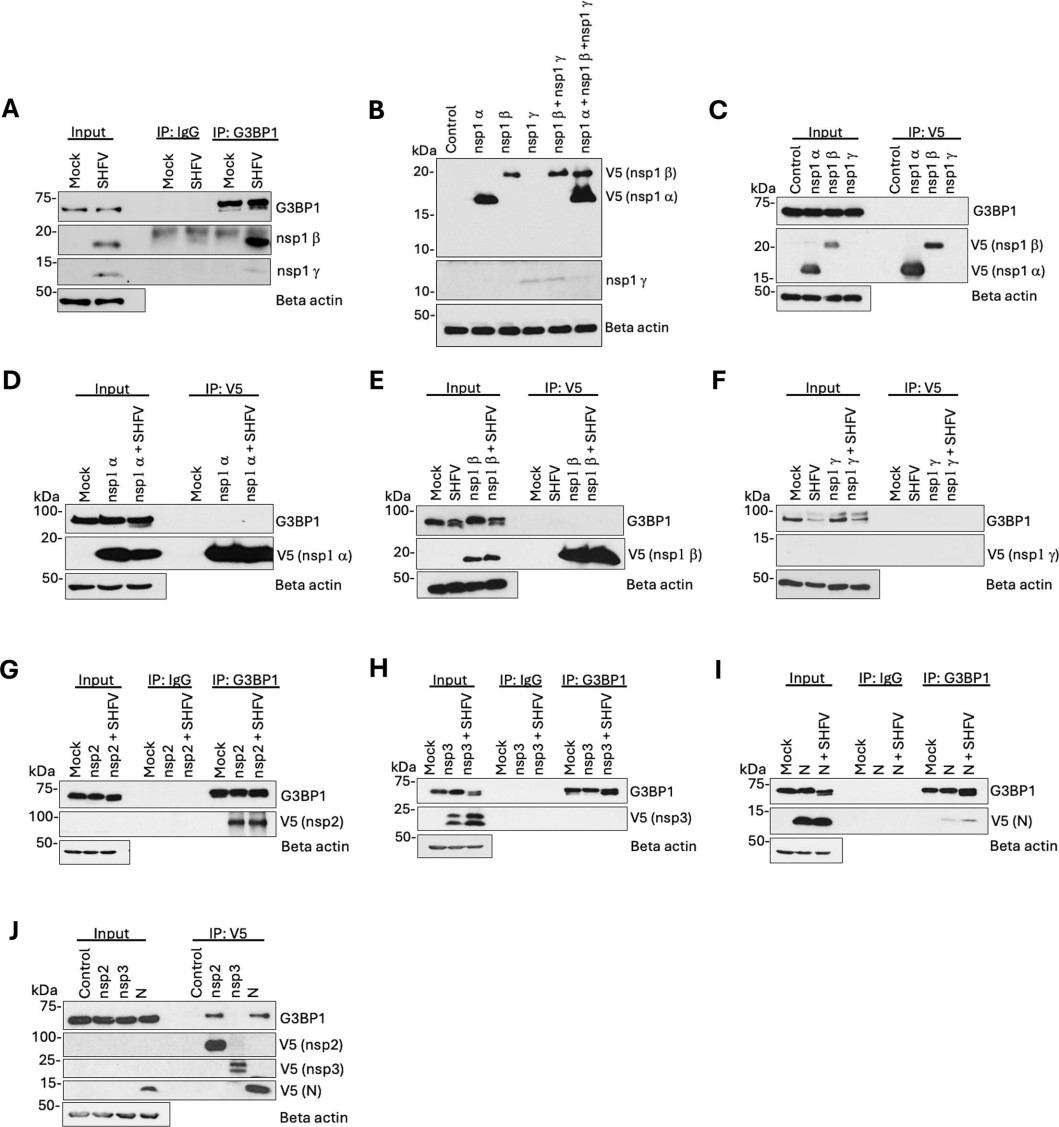
G3BP1 interacts with the SHFV nsp2 and nucleocapsid proteins but not with nsp1α, nsp1β, nsp1γ, or nsp3. (**A**) Whole-cell lysates were harvested in IP lysis buffer at 24 hpi from mock-infected or SHFV-infected (MOI of 0.1) MA104 cells. G3BP1 was immunoprecipitated using rabbit anti-G3BP1 antibodies. A rabbit anti-IgG antibody was used as an antibody control. Immunoprecipitated proteins were analyzed by WB using mouse anti-G3BP1 antibodies or rabbit anti-nsp1β or anti-nsp1γ antibodies. (**B and C**) MA104 cells were mock-transfected or transfected with mRNA encoding nsp1α, nsp1β or nsp1γ, each with a C-terminal V5 tag. (**B**) At 20 h post transfection (hpt), whole-cell lysates were harvested in 1× RIPA buffer and used for WB analysis with antibodies specific for V5 or nsp1γ. (**C**) At 20 hpt, whole-cell lysates were harvested in IP lysis buffer and the V5-tagged nsp1 proteins were immunoprecipitated using V5-trap magnetic agarose beads. Immunoprecipitated proteins were analyzed by WB using anti-G3BP1 and anti-V5 antibodies. (**D–F**) MA104 cells were mock-infected or infected with SHFV (MOI of 0.1) for 4 h, then independently transfected with mRNA encoding (D) nsp1α, (E) nsp1β, or (F) nsp1γ for 20 h. At 24 hpi, whole-cell lysates were immunoprecipitated using V5-trap magnetic agarose beads. Immunoprecipitated proteins were analyzed by WB using anti-G3BP1 and anti-V5 antibodies. (**G–I**) MA104 cells were mock-transfected or transfected with plasmid DNA encoding (**G**) nsp2, (**H**) nsp3, or (**I**) N protein for 48 h and then mock-infected or infected with SHFV (MOI of 0.1) for an additional 24 h. At 72 hpt, whole-cell lysates were immunoprecipitated with rabbit anti-G3BP1 antibody. Immunoprecipitated proteins were analyzed by WB using mouse anti-G3BP1 and mouse anti-V5 antibodies. (**J**) Whole-cell lysates prepared from MA104 cells that were mock-transfected or transfected with nsp2, nsp3, or N protein plasmid DNA for 72 h were immunoprecipitated using V5-trap magnetic agarose beads. Immunoprecipitated proteins were analyzed by WB using antibodies specific for G3BP1 and V5.

To determine whether G3BP1 interacts with SHFV nsp1α, nsp1β, or nsp1γ, we infected MA104 cells at an MOI of 0.1 for 24 h and then performed an immunoprecipitation assay on whole-cell lysates using anti-G3BP1 antibody followed by WB analysis of the precipitated proteins. Custom-made antibodies were used to detect nsp1α, nsp1β, or nsp1γ in the infected cell lysates. Consistent with the LC-MS/MS data, G3BP1 co-precipitated nsp1β and nsp1γ from infected lysates. However, nsp1α was not detected (Fig. 5A). The faint signal observed for nsp1γ and the absence of nsp1α may be due to weak ligand binding by the antibodies used in this assay. Also, we were unable to perform reciprocal co-immunoprecipitation assays using these antibodies. Therefore, we acquired synthetic mRNAs encoding the nsp1α, nsp1β, and nsp1γ proteins with a C-terminal V5 tag. Although nsp1α and nsp1β expression was detected by WB analysis using anti-V5 antibodies in transfected MA104 cell lysates after 20 h, we could only confirm nsp1γ expression with the custom nsp1γ antibody (Fig. 5B). To determine if either nsp1α, nsp1β, or nsp1γ reciprocally immunoprecipitated G3BP1, we independently transfected MA104 cells with each of the nsp1 mRNAs for 20 h, then used V5-trap magnetic agarose to perform immunoprecipitation on the whole-cell lysates followed by WB analysis. G3BP1 did not co-precipitate with any of the nsp1 proteins in mRNA-transfected cells (Fig. 5C). To determine if the nsp1 proteins could precipitate G3BP1 in the presence of an infection, we infected MA104 cells at an MOI of 0.1 for 4 h, then independently transfected nsp1α, nsp1β, or nsp1γ mRNA, and at 24 hpi immunoprecipitated whole-cell lysates with V5-trap magnetic agarose. Neither V5-tagged nsp1α, nsp1β nor nsp1γ co-precipitated G3BP1 from the transfected or infected and transfected cell lysates, indicating that the nsp1 proteins co-precipitated with G3BP1 due to being part of the viral replication complex (Fig. 5D through F).

To determine if G3BP1 interacts with either nsp2, nsp3, or the N protein, we transfected MA104 cells with plasmid DNA encoding either a V5-tagged nsp2, nsp3, or the N protein for 48 h. We then mock-infected or infected the cells with SHFV at an MOI of 0.1 for 24 h before performing immunoprecipitation on the whole-cell lysates with anti-G3BP1 antibodies. The low transfection efficiency of nsp2 (~30%) presented a challenge for protein detection in the input samples. However, a single band of the appropriate molecular weight was consistently detected in precipitated samples, confirming its expression (Fig. 5G). Both nsp2 and the N protein were individually co-precipitated by G3BP1 from both transfected and infected and transfected cell lysates (Fig. 5G and I). In contrast, nsp3 was not co-precipitated from either of these lysates (Fig. 5H). To determine if G3BP1 is reciprocally co-precipitated by either nsp2, nsp3, or the N protein, MA104 cells were independently transfected with a DNA construct encoding one of these proteins. At 72 h, whole-cell lysates were immunoprecipitated with V5-trap magnetic agarose, and the precipitates were analyzed by WB. G3BP1 was co-precipitated by both nsp2 and the N protein from transfected cell lysates, but not by nsp3 (Fig. 5J). These data indicate that nsp2 and the N protein are capable of forming a complex with G3BP1 and suggest that co-precipitation of the additional viral proteins with G3BP1 is due to their interaction within the RTC.

### SHFV nsp2 and nucleocapsid proteins expressed alone colocalize with G3BP1 foci

**Fig 6:**
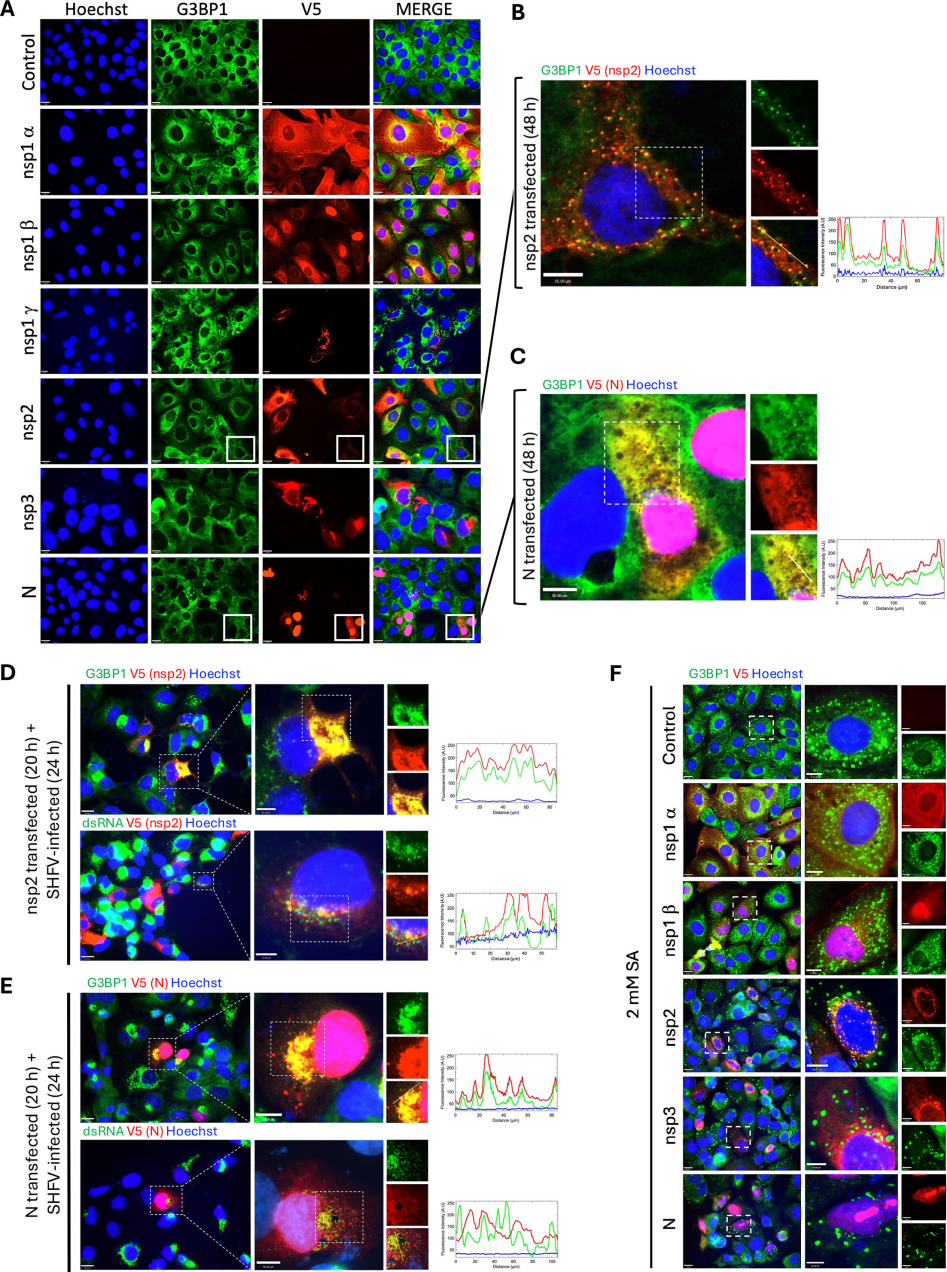
The SHFV nsp2 and N proteins colocalize with G3BP1 foci in infected or transfected cells. (**A**) MA104 cells were mock-transfected or transfected with mRNA encoding nsp1α or nsp1β with a C-terminal V5 tag for 20 h or transfected with plasmid DNA encoding nsp1γ, nsp2, nsp3, or N with a C-terminal V5 tag for 48 h. Cells were fixed, permeabilized, and incubated with antibodies specific for G3BP1 (green) and V5 (red). Nuclei were stained with Hoechst 33342 (blue). (**B**) Enlargement of a single nsp2-transfected cell from panel (A). Fluorescence intensity profiles of G3BP1 (green) and V5 (red) signals along the yellow cross-section line in the enlarged merged image are shown. (**C**) Enlargement of N-transfected cells from panel (A). Fluorescence intensity profiles of G3BP1 (green) and V5 (red) signals along the yellow cross-section line in the enlarged merged image are shown. (**D and E**) MA04 cells were infected with SHFV (MOI of 0.1) for 4 h and then mock-transfected or transfected with nsp2 or N plasmid DNA for 20 h. At 24 hpi, the cells were fixed, permeabilized, and incubated with antibodies specific for G3BP1 or dsRNA (green) and V5 (red). Nuclei were stained with Hoechst 33342 (blue). Fluorescence intensity profiles for G3BP1 or dsRNA (green) and V5 (red) signals along the yellow cross-section lines in the enlarged merged images are shown. (**F**) MA104 cells were mock-transfected or transfected with mRNA encoding nsp1α or nsp1β with a C-terminal V5 tag or transfected with plasmid DNA encoding nsp2, nsp3, or N with a C-terminal V5 tag. At 20 hpt, transfected cells were treated with 2 mM SA or 2 mM DTT for 45 min. Cells were fixed, permeabilized, and incubated with antibodies specific for G3BP1 (green) and V5 (red). Nuclei were stained with Hoechst 33342 (blue).

To determine the cellular localization of individual viral proteins and to detect their potential colocalization with G3BP1, MA104 cells were mock-transfected or transfected with mRNA encoding nsp1α or nsp1β for 20 h or transfected with DNA encoding nsp1γ, nsp2, nsp3, or N for 48 h, and then the cells were processed for IFA (Fig. 6A). We observed that nsp1α and nsp1γ were primarily located in the cytoplasm of transfected cells, while nsp1β was detected in both the nucleus and cytoplasm. Overexpression of nsp1γ appeared to be toxic to the cells, as a severe cytopathic effect was consistently observed in transfected cells at various times after transfection. None of the nsp1 proteins were observed to colocalize with G3BP1 foci in the transfected cells. The nsp2 and nsp3 proteins were detected primarily in the cytoplasm. In approximately 30% of the nsp2-transfected cells, we observed redistribution of G3BP1 into small foci that colocalized with nsp2 foci (Fig. 6B), indicating that nsp2 mediates recruitment of G3BP1 to perinuclear foci in the absence of other viral proteins. Colocalization of G3BP1 with nsp3 was not observed. The N protein was located mainly in the nucleus, with a faint signal also detected in the cytoplasm in areas that also contained G3BP1 (Fig. 6C).

To verify that nsp2 and the N protein localize to the sites of viral RNA replication and colocalize with G3BP1 in infected cells, MA04 cells were infected with SHFV at an MOI of 0.1 for 4 h, transfected with V5-tagged nsp2 or N plasmid DNA for 20 h, and then processed for IFA. Colocalization of plasmid-expressed nsp2 and G3BP1 (Fig. 6D, top panel) and of nsp2 and viral dsRNA (Fig. 6D, bottom panel) was observed in the infected cells. Plasmid-expressed N protein was detected primarily in the nucleus of the infected cells but was also observed to colocalize with G3BP1 in the cytoplasm (Fig. 6E, top panel). The cytoplasmic N protein localized to the same regions in the infected cells as the viral dsRNA (Fig. 6E, bottom panel). These data indicate that individually expressed nsp2 and N proteins colocalize with G3BP1 at the sites of SHFV RNA replication in the infected cells.

To determine whether the expression of an individual viral protein can inhibit SG assembly, MA104 cells were transfected with mRNA encoding nsp1α or nsp1β or transfected with DNA encoding nsp2, nsp3, or N for 20 h, incubated with SA (2 mM) or DTT (2 mM) for 45 min and then the cells were processed for IFA. None of the individually expressed viral proteins inhibited the assembly of SGs induced by either exogenous SG inducer (Fig. 6F).

### The nsp2 G3BP1 binding motif, FGAP, is conserved in simian arteriviruses

**Fig 7:**
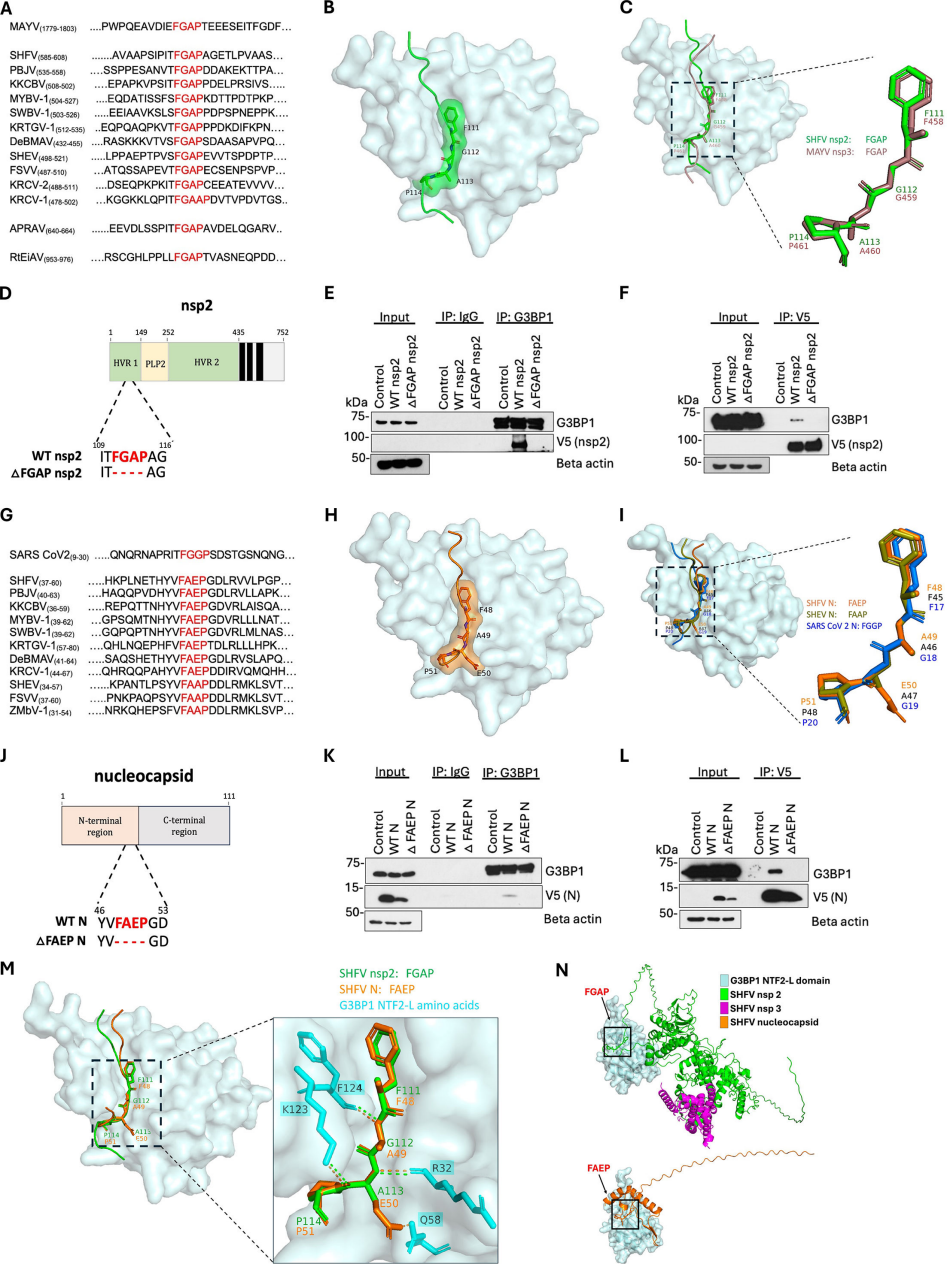
SHFV nsp2 binds G3BP1 via a FGAP motif, whereas SHFV N binds G3BP1 via a noncanonical FAEP motif. (**A**) Alignment of the FGAP-containing amino acid sequence of MAYV nsp3 and of the nsp2s of 11 simian arteriviruses and 2 non-simian arteriviruses. (**B and C**) Predicted model of SHFV nsp2 (aa 106–116) (green) in complex with the hydrophobic pocket of the *Chlorocebus sabaeus* G3BP1 NTF2-L domain (aa 1–140) (cyan) generated by AlphaFold 3. SHFV nsp2-G3BP1 model pTM score = 0.32, ipTM score = 0.77 (**C**) Structural alignment of the SHFV nsp2 (green) and MAYV nsp3 (aa 453–463) (brown) FGAP motifs docked in the hydrophobic pocket of the G3BP1 NTF2-L domain (cyan). Mayaro virus nsp3-G3BP1 model pTM score = 0.33, ipTM score = 0.85. (**D**) Schematic representation of SHFV nsp2 domain organization and an FGAP deletion mutant. HVR 1- hypervariable region 1; PLP, papain-like protease; Black Bars, transmembrane domains. (**E and F**) Whole-cell lysates from MA104 cells transfected with either WT nsp2 or ∆FGAP nsp2 mutant plasmid DNA were harvested in IP lysis buffer at 72 hpt and immunoprecipitated with (**E**) rabbit anti-G3BP1 antibody or (**F**) V5-trap magnetic agarose beads. Immunoprecipitated proteins were analyzed by WB using mouse anti-G3BP1 and mouse anti-V5 antibodies. (**G**) Alignment of the ITFGGP-containing sequence of SARS-CoV 2 N protein with the FAEP (or FAAP) containing sequences of the N proteins of 11 simian arteriviruses. (**H and I**) Predicted model of SHFV N protein (aa 43–56) (orange) in complex with the hydrophobic pocket of the G3BP1 NTF2-L domain (cyan) generated by AlphaFold 3. SHFV N protein-G3BP1 model pTM score = 0.56, ipTM score = 0.35. (**I**) Structural alignment of the FAEP (orange) and FAAP (aa 40–53) (olive green) motifs in simian arterivirus N proteins and the ITFGGP motif in the SARS-CoV 2 N protein (aa 12–22) (blue) docked in the hydrophobic pocket of the G3BP1 NTF2-L domain (cyan). SHEV N-G3BP1 model pTM score = 0.54. ipTM score = 0.14, SARS CoV2 N-G3BP1 (PDB:7SUO) (**J**) Schematic representation of the SHFV N protein domain organization and the FAEP deletion mutant. (**K and L**) MA104 cells were transfected with either N protein or ∆FAEP plasmid DNA. Cells were harvested in IP lysis buffer at 72 hpt and immunoprecipitated with (**K**) rabbit anti-G3BP1 antibody or (**L**) V5-trap magnetic agarose beads. Immunoprecipitated proteins were analyzed by WB using mouse anti-G3BP1 and mouse anti-V5 antibodies. (**M**) Close-up view of the predicted interactions between the nsp2 FGAP motif (green) or the N protein FAEP motif (orange) and amino acids in the G3BP1 NTF2-l domain hydrophobic pocket. (**N**) The amino acid sequences of the G3BP1 NTF2-L domain and of the SHFV nsp2, nsp3 and N proteins were entered into the AlphaFold3 program to generate predicted protein structures. The generated models were analyzed using PYMOL software. G3BP1 NTF2 domains (cyan), nsp2 (green), nsp3 (pink), and the N protein (orange). The interfaces between FGAP and G3BP1 and between FAEP and G3BP1 are indicated by black boxes. SHFV nsp2- nsp3-G3BP1 model pTM score = 0.27, ipTM score = 0.28.

We further investigated the interactions between G3BP1 and nsp2, and G3BP1 and N, by searching for possible binding sites in these viral proteins. A repeated FGDF or FGxF-like motif located in the nsp3 hypervariable domain (HVD) of the Old World alphaviruses was previously shown to bind in the conserved hydrophobic pocket located in the NTF2-L domain of G3BP1 (32, 39–41). A subsequent study showed that a non-canonical G3BP1 binding motif, FGAP, found in the nsp3 of MAYV, another alphavirus, also functioned as a binding motif for G3BP1 (42). To determine whether a known G3BP1 binding motif was present in any of the SHFV proteins, we searched the SHFV genome coding regions and discovered a single FGAP motif located in the hypervariable region (HVR) of nsp2. Sequence alignment of this nsp2 region for all arterivirus genomes showed that the FGAP motif was also present in the same nsp2 region of nine other known simian arteriviruses, as well as of the African pouched rat arterivirus (APRAV) and the RtEiAV arterivirus (Fig. 7A). However, this motif was not found in the genomes of LDV, EAV, PRRSV-1 or PRRSV-2. An *in silico* interaction model of G3BP1 and SHFV nsp2, generated with AlphaFold 3, predicted that the nsp2 FGAP motif fits well into the hydrophobic pocket of the NTF2-L domain of G3BP1 (Fig. 7B). Structural alignment of the MAYV nsp3 and SHFV nsp2 motif models showed that these two FGAP motifs are predicted to bind the G3BP1 NTF2-L hydrophobic pocket in a similar conformation (Fig. 7C).

To determine whether the SHFV nsp2 FGAP motif is necessary for interaction with G3BP1, we constructed a DNA plasmid encoding an nsp2 FGAP motif deletion mutant (∆FGAP nsp2) (Fig. 7D). MA104 cells were mock-transfected or transfected with the nsp2 wild type (WT) or the ∆FGAP mutant plasmid DNA for 72 h. Whole-cell lysates were prepared and immunoprecipitated using either anti-G3BP1 antibody or V5-trap magnetic agarose, followed by WB analysis. Consistent with our previous experiments (Fig. 5G), the WT nsp2 co-precipitated with G3BP1, but the ∆FGAP nsp2 mutant did not (Fig. 7E). In the reciprocal immunoprecipitation assay with V5-trap magnetic agarose, G3BP1 co-precipitated with the WT nsp2, but did not co-precipitate with the ∆FGAP nsp2 mutant (Fig. 7F). These data confirm that the FGAP motif found in the SHFV nsp2 is required for interaction with G3BP1 and is highly conserved among the simian arteriviruses.

### The SHFV N protein binds G3BP1 via a non-canonical FAEP motif

Neither an FGxF-like nor an FGAP motif was identified in the SHFV N protein sequence, yet an interaction with G3BP1 was observed, suggesting that an unidentified interaction motif existed in the SHFV N protein. Recent crystallography data showed that the SARS-CoV-2 N protein binds to the NTF2-L domain of G3BP1 via a non-canonical ITFGGP motif (36, 43), which is more similar to the MAYV FGAP motif (42). A sequence analysis of the SHFV N protein identified an FAEP motif that was conserved in the N proteins of seven of the known simian arteriviruses. An FAAP motif was found in the N protein of three additional simian arteriviruses. These motifs were not present in the N proteins of any other arteriviruses (Fig. 7G). An AlphaFold 3 model of the SHFV N protein and G3BP1 interaction predicted that the N FAEP motif fits in the hydrophobic pocket of the G3BP1 NTF2-L domain (Fig. 7H). The FAAP motif was also predicted to bind the hydrophobic pocket of the G3BP1 NTF2-L domain in a similar manner. Structural alignment of the SHFV N, SHEV N, and SARS-CoV-2 N (PDB: 7SUO) motifs showed that they are structurally similar, supporting the hypothesis that the SHFV FAEP motif can bind the NTF2-L hydrophobic pocket (Fig. 7I).

To determine whether the predicted SHFV FAEP motif mediates interaction with G3BP1, we constructed a DNA plasmid encoding the N protein with a deleted FAEP motif (∆FAEP N) (Fig. 7J). Whole-cell lysates prepared from MA014 cells that were mock-transfected or transfected with the WT N protein or the ∆FAEP mutant plasmid DNA for 72 h were immunoprecipitated with either anti-G3BP1 antibody or V5-trap magnetic agarose and then analyzed by WB. We observed decreased expression of the ∆FAEP N protein even though all experimental conditions were consistent for both plasmids. The WB analysis showed that the WT N protein co-precipitated with G3BP1, but the ∆FAEP N protein did not (Fig. 7K). In the reciprocal V5 immunoprecipitation assay, G3BP1 co-precipitated with the WT N protein but not with the ∆FAEP N protein (Fig. 7L). These data identify FAEP as an SHFV non-canonical G3BP1 binding motif and further indicate that SHFV utilizes a unique motif that is conserved among simian arteriviruses as a second mode of interaction with G3BP1.

Finally, we performed a structural alignment of the models for the SHFV nsp2 FGAP and the N protein FAEP motifs and observed that both motifs appear structurally similar and fit well into the NTF2-L hydrophobic pocket. Both motifs are predicted to interact with key G3BP1 residues such as Arg-32, Lys-123, and Phe-124 (Fig. 7M). An *in silico* model generated by AlphaFold 3 shows that both the N protein and nsp2 could interact with individual G3BP1 NTF2-L domains (Fig. 7N). The known interaction of nsp3 with the C-terminus of nsp2 is consistent with its abundant co-precipitation with G3BP1 (Fig. 4A).

### G3BP1 is cleaved in SHFV-infected cells

**Fig 8:**
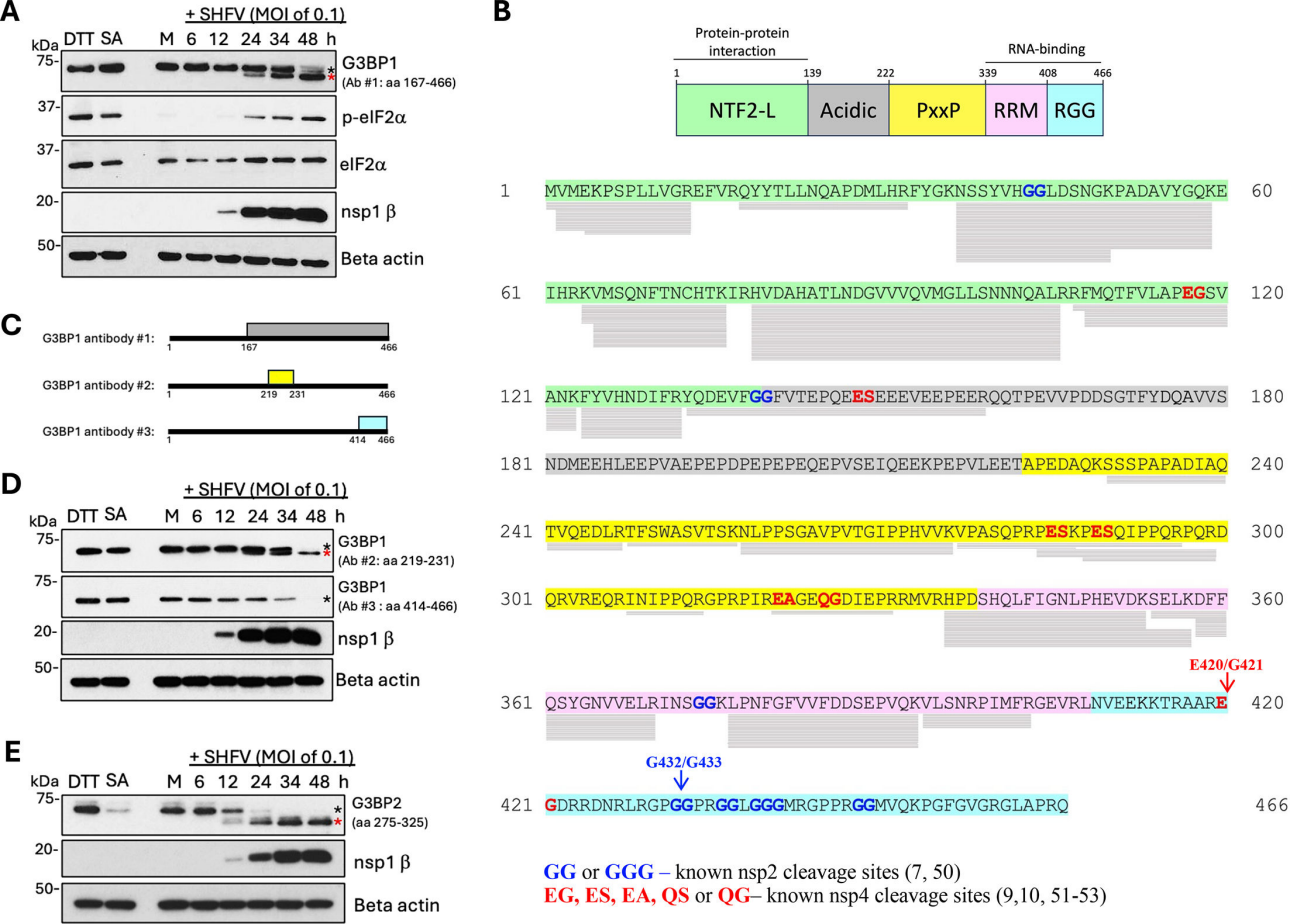
G3BP1 and G3BP2 are cleaved in SHFV-infected cells. (**A**) MA104 cells were mock-infected or infected with SHFV (MOI of 0.1). At 24 h, mock-infected cells were incubated with DTT (2 mM) or SA (2 mM) for 45 min. Whole-cell lysates harvested at different times after infection were analyzed by WB using antibodies specific for G3BP1, p-eIF2α, eIF2α, or SHFV nsp1β. (**B**) Mapped G3BP1 trypsin peptides detected by LC-MS/MS. G3BP1 domain sequences are indicated. NTF2-L, nuclear transport factor 2-like domain (green); Acidic, acidic-rich region (gray); PXXP, Proline-rich region (yellow); RRM, RNA recognition motif (pink); RGG, Arg-Gly-rich region (light blue). Gray bars under the G3BP1 sequence indicate the mapped locations of unique peptides detected by LC-MS/MS in SHFV-infected cells. The locations of known nsp2 PLP cleavage sites (GG, blue) and of known nsp4 SP cleavage sites (EG, EA, ES and QG, red) are indicated. The red arrow indicates a possible site of G3BP1 cleavage by nsp4 SP, and the blue arrow indicates a possible site of cleavage by nsp2 PLP2 in the C-terminus of G3BP1. (**C**) Schematic representation of the G3BP1 peptides used to make the three different G3BP1-specific antibodies tested in the WB analyses. (**D and E**) Whole-cell lysates harvested at different times after infection were analyzed by WB using (**D**) two different antibodies specific for G3BP1 or (**E**) an antibody specific for G3BP2. Black asterisk—full-length G3BP. Red asterisk—cleaved G3BP.

We next analyzed G3BP1 levels by WB to determine whether G3BP1 expression levels were modified in the infected cells. MA104 cells were mock-infected or infected with SHFV at an MOI of 0.1, and cell lysates were harvested at different times after infection for WB analysis using G3BP1 Ab #1. Replicate mock-infected cells were also treated with SA or DTT to induce SGs. While G3BP1 migrated as a ~65 kDa band in uninfected cell lysates, we consistently detected an additional faster migrating band of ~58 kDa in the infected cell lysates by 24 hpi. The ~65 kDa band was observed to decrease, and the ~58 kDa band to increase in intensity over time, and this timing correlated with increased viral protein production as indicated by the nsp1β levels (Fig. 8A). Viral proteases have been previously shown to cleave G3BP1 in infected cells to inhibit SG formation (44–48). Alternatively, a porcine epidemic diarrhea virus infection was shown to induce G3BP1 cleavage by the cellular caspase 8 protease to achieve SG inhibition (49). To identify possible G3BP1 cleavage sites in the infected cells, we aligned the G3BP1 peptides detected in the SHFV-infected cells by LC-MS/MS and searched for peptides cleaved at known arterivirus PLP sites (GG or GGG) (7, 50) or at known nsp4 SP cleavage sites (EG, ES, EA, QS, or QG) (9, 10, 51–53) (Fig. 8B). The observed size of the G3BP1 cleavage product suggests a small fragment was cleaved from the N- or C-terminus. Several trypsin peptides that matched the N-terminal NTF2-L domain of G3BP1 covered the putative viral protease cleavage sites in this region. However, no peptides from the C-terminal RGG domain were detected. A nsp4 SP cleavage site (E420/G421) and several possible PLP cleavage sites (G432/G433, G436/G437, and G439/G440/G441) are located in the C-terminal region of G3BP1. Cleavage at either E420/G421 by nsp4 SP or at G432/G433 or G436/G437 by the nsp2 PLP is predicted to produce an N-terminal G3BP1 cleavage product of the approximate size of the observed band.

The G3BP1 peptide used to produce the antibody (Ab #1) used in the initial experiments (Fig. 8A) consisted of amino acids 167–466. This antibody can detect the full-length G3BP1 protein as well as ones with either the N- or C-terminus removed (Fig. 8C). To confirm that the two bands detected were G3BP1-specific, a second antibody (Ab #2) produced to a peptide consisting of amino acids 219–231 that can also detect the full-length protein as well as ones that had been cleaved at either the N- or C-terminus was used for WB analysis of the infected MA104 cell lysates (Fig. 8C). The two bands of the same sizes were detected by Ab #2 and by Ab #1. As observed with Ab #1, the intensity of the ~65 kDa G3BP1 band decreased, while the intensity of the ~58 kDa G3BP1 band increased with time after infection (Fig. 8D). To further investigate whether G3BP1 is cleaved at a site near its C-terminus, an additional G3BP1 antibody (Ab #3) made to amino acids 414–466 that would detect the full-length G3BP1 protein but not one with a cleaved C-terminus was used for WB analysis. A single G3BP1 band, migrating at ~65 kDa was detected by G3BP1 Ab #3, and the intensity of this band decreased over time, providing further evidence that G3BP1 is cleaved at a site in the C-terminal RGG domain (Fig. 8D).

The G3BP family consists of three homologous proteins, G3BP1 and two splice variants of G3BP2, G3BP2a (482 aa) and G3BP2b (449 aa) (54). The G3BP proteins share a similar domain architecture and are known to form homodimers or heterodimers via their N-terminal NTF2-L domains. Both G3BP1 and G3BP2 were shown to play important roles in SG formation (22). To determine whether G3BP2 is also cleaved in SHFV-infected cells, a G3BP2-specific antibody, produced to a peptide consisting of amino acids 275–325 was utilized for WB analysis of infected MA104 cell lysates. A G3BP2 band migrating at ~68 kDa was detected in mock-infected cell lysates and at early times in the infected cell lysates. The intensity of the ~68 kDa band decreased over time, while a faster migrating band of ~50 kDa, that was observed by 12 hpi, increased in intensity throughout the course of the infection (Fig. 8E). Several putative nsp2 PLP cleavage sites are located in the C-terminal region of the G3BP2 protein sequence. These data indicate that both G3BP1 and G3BP2 are cleaved in SHFV-infected cells and suggest that G3BP cleavage is mediated by a viral protease. However, additional studies are needed to identify the viral protease and the cleavage sites in the two G3BPs.

## DISCUSSION

Many studies have shown that viral infections trigger the cellular SG response, primarily through activation of the stress kinases, PERK and/or PKR (55). Activation of either kinase results in the inhibition of global cap-dependent translation through the phosphorylation of eIF2α, which leads to the assembly of translationally silenced mRNPs known as SGs. SGs typically have an antiviral effect since they contain translation initiation molecules and proviral components that are needed for viral protein translation. To overcome the antiviral effects of SGs, many viruses express effector proteins that manipulate steps or components of the stress response pathway to inhibit SG assembly (48, 56).

Despite observing PKR activation and increased levels of eIF2α phosphorylation by 24–48 hpi, *bona fide* SGs were not detected at any time in SHFV-infected MA104 cells. However, the SHFV infection did not inhibit the formation of SGs induced by exogenous inducers, but the number of SGs induced was significantly reduced compared to the number induced in uninfected cells. In contrast, the arterivirus PRRSV-1 was recently shown to replicate in the presence of *bona fide* SGs that were induced through PERK/eIF2α activation (27), suggesting that members of the arterivirus family have evolved different strategies to counteract the SG response. A similar diversity in the viral response to SG formation was observed among the coronaviruses, which are also members of the order *Nidovirales* (26, 30, 55).

Virus infections that interfere with the assembly of SGs often do so by redistribution, sequestration, or cleavage of SG components. G3BP1, the primary nucleator of SGs, is a multifunctional protein reported to have roles in cell-cycle regulation, mRNA metabolism, ribosomal quality control, and the immune response, as well as SG assembly (26, 57). During SG assembly, G3BP1 is reported to function as a “scaffold,” mediating protein-protein and protein-RNA interactions. G3BP1 is targeted by diverse virus families to subvert SG formation and is sometimes also utilized as a proviral replication factor (26). A murine norovirus (MNV) infection was reported to inhibit SG formation, but foci containing G3BP1 but not eIF3B were observed near the viral replication sites (58, 59). We observed a similar redistribution of G3BP1 and other SG-related proteins (G3BP2, TIA-1, Caprin-1, and USP10), but not of translation initiation proteins (eIF4G and eIF3A) or small ribosomal proteins (rpS6), to sites of viral replication. Similar to the foci seen in MNV-infected cells, the SHFV-induced G3BP1 foci were stable in the presence of CHX, unlike the dynamic SGs induced by SA. G3BP1 was determined to function as a proviral host factor for MNV replication and also for hepatitis C virus, ZIKV, CHIKV, and SFV. Whether G3BP1 also provides a proviral function for SHFV replication remains to be investigated.

Recruitment of SG-related proteins through interaction with a viral protein is one means utilized by several viruses to subvert SG assembly. Tandem FGDF or FGxF-like motifs located in the nsp3 of several Old World alphaviruses were reported to bind a hydrophobic groove in the NTF2-L domain of G3BP1 (32, 39–41, 60), and an FGAP motif located in the nsp3 of MAYV was later identified as a non-canonical G3BP1 binding motif (42). An ITFGGP sequence located in the N protein of SARS CoV2 was also shown to bind in the hydrophobic groove of the G3BP1 NTF2-L domain (31, 36, 43). Here, we showed colocalization by IFA and interaction by reciprocal co-immunoprecipitation assays of the SHFV nsp2 and N proteins with G3BP1 in transfected or transfected and infected cells. Additionally, we showed that a single FGAP motif located in nsp2 and a unique FAEP motif located in the N protein of SHFV were required for interaction with G3BP1. Unlike the alphaviruses, which require tandem motifs to efficiently interact with G3BP1, a single motif in either the SHFV nsp2 or the N proteins was sufficient to mediate interaction with G3BP1. Other proteins, such as the SARS-CoV-2 N protein and the cellular proteins, Caprin-1 and USP10, which are known G3BP1 interactors, also contain a single G3BP1 binding motif (23).

G3BP1 was shown to function as a replication factor for Old World alphaviruses through interaction with the viral nsp3 (61). The role of G3BP1 in SHFV life cycle may differ depending on its interaction with each of the two viral proteins. Given that G3BP1 interacts with SHFV nsp2, localizes to the sites of viral replication, and co-precipitated multiple nsps of the RTC, it is plausible that G3BP1 also functions as a replication factor for SHFV. The arterivirus N protein is not required for viral replication but was shown in EAV-infected cells to form a filamentous network of tubules near the sites of viral replication (37, 38) and to be required for encapsidation of the viral genome. An RNA binding domain in the N-terminal region of the N protein facilitates interaction with genomic RNA, and a capsid binding domain in the C-terminal region enables the formation of N protein homodimers (62). A preformed nucleocapsid buds into the ER or Golgi membranes where the viral structural proteins are localized (2). Several additional structural proteins were found to co-precipitate with G3BP1 in SHFV-infected cells, suggesting that G3BP1 may also function as an RNA chaperone for virion assembly through its interaction with the N protein.

Among arterivirus proteins, nsp2 has the largest sequence variation due to the presence of two HVRs (50, 63). The FGAP motif, which is located in a SHFV nsp2 HVR, was conserved in the nsp2 coding region of nine of the known simian arteriviruses, but was not present in the EAV, LDV or PRRSV-1/2 nsp2 proteins. Similarly, the simian arterivirus N proteins have conserved FAEP or FAAP motifs while other arterivirus N proteins do not, further supporting the evolutionary divergence of the simian arteriviruses from other members of the arterivirus family. Further studies are required to confirm that the divergent nsp2 and N proteins of other simian arteriviruses can also interact with G3BP1 via their conserved motifs.

While nsp1β of PRRSV-1 was shown to co-precipitate with G3BP1 from infected or transfected MARC-145 cell lysates, evidence supporting a direct interaction between nsp1β and G3BP1 was not presented (35). Our LC-MS/MS data showed that multiple viral proteins co-precipitated with G3BP1 from SHFV-infected MA104 cell lysates. Although nsp1β and nsp1γ co-precipitated with G3BP1 from infected cell lysates, neither of these proteins co-precipitated G3BP1 in a reciprocal co-immunoprecipitation assay. Interestingly, in an unstructured region of the nsp1γ protein, we identified an FGAF motif sequence, similar to the alphavirus FGxF motifs. However, introducing alanine mutations into the FGAF motif of nsp1γ in a full-length infectious clone virus did not prevent the co-precipitation of nsp1γ with G3BP1 and did not inhibit the redistribution of G3BP1 to foci in the infected cells (data not shown). Furthermore, the FGAF motif of SHFV nsp1γ was not predicted to bind the hydrophobic pocket of the G3BP1 NTF2-L domain in an *in silico* interaction model of G3BP1 and SHFV nsp1γ generated with AlphaFold 3. In addition to the nsp1s, nsp3, the second most abundant protein that co-precipitated with G3BP1, could not be verified as an interacting partner of G3BP1 in reciprocal co-immunoprecipitation assays from lysates of transfected or transfected and infected cells. It is likely that nsp3 was so abundantly co-precipitated with G3BP1 due to its known interaction with nsp2 (11, 13, 52, 64). An *in silico* model generated by AlphaFold 3 provided a plausible orientation of the interaction between these proteins, in which the N and/or nsp2 proteins interact with individual G3BP1 NTF2-L domains and nsp3 interacts with the C-terminal of nsp2. These data strongly suggest that the co-precipitation of nsp1α, nsp1β, and nsp1γ and nsp3 with G3BP1 was due to their interaction with other viral proteins in the RTC.

Several studies have found that SG assembly can be inhibited by viral protease cleavage of G3BPs (44–47, 65). Members of the G3BP family, G3BP1 and splice variants, G3BP2a and G3BP2b, share similar domain organization and function in SG nucleation (54). The N-terminal NTF2-L domain mediates G3BP homodimer or heterodimer protein-protein interactions, and also interactions with other cellular and viral proteins, whereas the C-terminal RGG domain mediates interactions with mRNA and 40s ribosomal subunits (23). Our data indicate that cleavage of both G3BP1 and G3BP2 occurs in SHFV-infected cells. The sizes of the G3BP1 and G3BP2 cleavage products differ, suggesting that the locations of the cleavage sites in the two proteins are not identical.

G3BP cleavage products were previously observed in cells infected with foot-and-mouth disease virus (FMDV), equine rhinitis A virus (EARV) (47), or feline calicivirus (FCV) (45). PLP L^pro^ of FMDV and EARV was reported to cleave G3BP1 and G3BP2 at unknown sites near the C-terminal RRM domain or RGG domain (47). FCV SP NS6^pro^ cleaves G3BP1 in the RRM domain at E405 (45). Putative cleavage sites for both the SHFV nsp2 PLP and the nsp4 SP are located in the G3BP1 protein sequence. The PLP domain of arterivirus nsp2s was shown to cleave in *cis* and *trans* at GG sites (50, 63), and arterivirus nsp4 SPs were shown to cleave at either EG, ES, EA, QS, or QG sites (9, 10, 52, 53). The peptide mapping data suggest that G3BP1 is cleaved near the C-terminal RGG domain. Cleavage at only the G43/G44 site in the N-terminus or either at E420/G421, or G432/G433 or G436/G437 site in the C-terminus would produce a G3BP1 cleavage product of the detected size in the SHFV-infected cell lysates. The modeling data indicated that the interaction site in G3BP1 for the two SHFV proteins is located in the N-terminus NTF2-L domain, and the LC-MS/MS analysis of G3BP1 peptides obtained from SHFV-infected cells detected several noncleaved peptides mapping in the region of the G43/G44 site in the N-terminus. Both the nsp2 PLP and the nsp4 SP cleavage sites are located in the G3BP1 RGG domain, and a cleavage product was not detected by G3BP1 Ab #3, which targeted the C-terminal RGG domain. We did not detect G3BP1 cleavage in nsp2-transfected cells, and no cellular protein targets of any arterivirus nsp2 PLPs have yet been reported. Although G3BP cleavage by the nsp2 PLP is plausible, it is also possible that nsp2 cooperates with the nsp4 SP to cleave G3BP1. The PRRSV nsp2 was shown to act as a cofactor for nsp4 SP cleavage activity (52, 66, 67). Both the PRRSV and EAV nsp4 SPs were shown to cleave multiple sites in the cellular protein, NF-κB essential modulator (NEMO), in infected cells (68, 69), further supporting the hypothesis that the SHFV nsp4 SP could be responsible for cleavage of G3BP1 C-terminus. Alternatively, an SHFV infection could activate a cellular protease to cleave G3BP1.

In addition to its protease activity, the PLP domain in SHFV, LDV, PRSSV, and EAV nsp2 was reported to function as a deubiquitinating enzyme (DUB) and was shown to remove K63-linked polyubiquitin from RIG-I (70). Furthermore, the PLP domain of PRRSV nsp2 was shown to remove K48-linked polyubiquitin from IkBα (71). K63-linked polyubiquitination of G3BP1 by TRIM21 and TRIM25 was shown to be involved in the disassembly of SGs induced by oxidative stress or heat shock, but not PRK-induced SGs (72–74). Whether the SHFV nsp2 PLP domain is involved in G3BP1 deubiquitination is not known.

Here, we provide evidence that an SHFV infection counteracts *bona fide* SG assembly through several mechanisms downstream of eIF2α activation. SG proteins, including the key SG nucleating proteins G3BP1 and G3BP2, are redistributed to sites of viral replication. G3BP1 is recruited to the viral replication sites through interaction with the SHFV nsp2 and N proteins. These viral proteins compete with the cell protein interaction partners of G3BP1, changing the G3BP1 interactome. Cleavage of the G3BP1 and likely also of the G3BP2 C-terminal domains at yet unknown sites prevents G3BP interaction with mRNA and translation initiation components including the 40S ribosomal subunit, which are required for SG assembly. Some but not all of the G3BP is recruited to the areas of viral replication and is cleaved in a time-dependent manner in SHFV-infected cells, resulting in a reduced number of SGs induced by treatment of infected cells with an exogenous inducer. Further studies are needed to determine whether G3BP proteins play proviral roles during the SHFV replication cycle.

## MATERIALS AND METHODS

### Cells and virus

The MA104 cell line was obtained from American Type Culture Collection (ATCC). MA104 cells are embryonic African green monkey epithelial kidney cells. These cells were cultured at 37°C in a 5% CO_2_ atmosphere using 1× Dulbecco’s modification of Eagle’s medium (Corning, #15-013-CV) supplemented with 1% l-glutamine, 7.5% fetal bovine serum (R&D Systems, #S11150), and ~1% Pen/Strep (Gibco, #15140-122).

Whole blood samples of rhesus macaque monkeys were obtained from Yerkes Regional Primate Center. Peripheral blood mononuclear cells were isolated using SepMate-50 tubes (Stemcell Technologies) and Lymphocyte separation media (Corning, #25-072-CV) by density gradient centrifugation. Isolated monocytes were resuspended in monocyte attachment media (Promocell, #C28051) and seeded in 24-well plates at a density of ~10^6^ cells/well. Following a 2 h attachment period, monocytes were gently washed with RPMI-1640 media, then incubated with RPMI-1640 media supplemented with 1% autologous donor serum, 10% heat-inactivated FBS (Atlas Biologicals), ~1% Pen/Strep (Gibco, #15140-122), and 25 ng/mL recombinant human macrophage colony stimulating factor (Perprotech, #AF-300-25). Differentiating macrophages were cultured at 37°C in a 5% CO_2_ atmosphere for 8 days and two-thirds of the culture media was replaced with fresh media every 3 days.

An aliquot of SHFV LVR 42-0/M6941 was obtained from ATCC. The virus was sequentially plaque-purified three times and then amplified once on MA104 cells (MOI of 0.01) to produce a stock pool. To make experimental virus pools, MA104 monolayers were infected with the stock virus at an MOI of 0.2, and the culture fluid was harvested at 32 hpi. The infectivity titer of the clarified culture fluid was determined to be 1 × 10^7^ PFU/mL by plaque assay on MA104 cells and was aliquoted and stored at −80°C.

MA104 cells or macaque macrophages were infected at an MOI of 0.1 or 1. Virus adsorption was allowed for 1 h at 37°C in a 5% CO_2_ atmosphere, then the inoculum was removed, and cells were washed three times with Hank’s balanced salt solution. Infected cells were then incubated with cell-specific medium at 37°C in a 5% CO_2_ atmosphere until harvest at indicated times.

### Construction of DNA plasmids and DNA transfection

The SHFV sequences for the nsp1γ, nsp2, nsp3 or N protein coding regions, each with a V5 tag (GKPIPNPLLGLDST) fused at the C-terminus, were used to order gBlocks Gene Fragments from Integrated DNA Technologies (IDT). The nsp1γ gBlock was cloned into the pEF6V5-HIS-TOPO plasmid (Thermo Fisher Scientific, #K961020) using flanking BamHI and XbaI restriction sites, and nsp2, nsp3, and the N protein gBlocks were cloned into the pcDNA 3.1+ plasmid (Thermo Fisher Scientific, #V79020) using flanking SpeI and XbaI restriction sites. Plasmids were transformed into XL Ultra competent cells (Agilent Technologies) using the manufacturer’s protocol. Five colonies from each transformation were randomly picked from plates and grown overnight in liquid culture (LB media plus 50 µg/mL kanamycin or 50 µg/mL carbenicillin). Plasmid DNAs were extracted from liquid cultures, purified, and sequence verified by Sanger sequencing (Psomagen).

The nsp2 FGAP and N FAEP deletion mutants were generated by site-directed mutagenesis using a QuickChange Lightning Site Directed Mutagenesis Kit (Agilent, #210518) according to the manufacturer’s protocol. The primers used for the ∆FGAP mutant were 5′-CAGGGTCTCTCCTGCCGTGATCGGTATGG-3′ and 5′-CCATACCGATCACGGCAGGAGAGACCCTG-3′ and for the ∆FAEP mutant were 5′-GCCTCTAAATGAGACACATTATGTTGGCGACCTCCGAG-3′ and 5′-CTCGGAGGTCGCCAACATAATGTGTCTCATTTAGAGGC-3′.

Subconfluent MA104 monolayers seeded on coverslips in a 24-well plate were transfected with 1 µg of plasmid DNA per well. Cells seeded in a 10 cm dish were transfected with 10 µg of plasmid DNA using X-treme GENE 360 Transfection Reagent (Millipore Sigma, #8724105001) according to the manufacturer’s protocol.

### Construction of nsp1 mRNA and mRNA transfections

All of the mRNAs used in this work were designed and synthesized by *in vitro* transcription reactions. Briefly, plasmids (GenScript) were linearized with NotI-HF (NEB) overnight at 37°C, purified by sodium acetate (Thermo Fisher Scientific) precipitation, and rehydrated with nuclease-free water. *In vitro* transcription was performed overnight at 37°C using the HiScribe T7 Kit (NEB) following the manufacturer’s instructions (N1-methyl-pseudouridine modified). The resulting RNA was treated with DNase I (Aldevron) for 30 min to remove the template and then purified using lithium chloride precipitation (Thermo Fisher Scientific). The RNA was heat denatured at 65°C for 10 min before capping with a Cap-1 structure using guanylyltransferase and 2′-O-methyltransferase (Aldevron). mRNA was then purified by lithium chloride precipitation, treated with alkaline phosphatase (NEB), and purified again. The mRNA concentration was measured using a NanoDrop instrument. Purified mRNA products were analyzed by gel electrophoresis to ensure purity.

MA104 monolayers seeded on coverslips in a 24-well plate were transfected with 250 ng of mRNA per well. Cells seeded in a 10 cm dish were transfected with 3 µg of mRNA using Lipofectamine MessengerMax Transfection Reagent (Thermo Fisher Scientific, #LMRNA001) according to the manufacturer’s protocol.

### Chemicals

SA (#S7400-100G) and CHX (#239763M) were obtained from Sigma-Aldrich. DTT (#R0861) was obtained from Thermo Fisher Scientific.

### IFA

Sub-confluent monolayers of macaque MΦ or MA104 cells grown on 15 mm glass coverslips in a 24-well plate were either mock-infected, transfected, or infected and transfected. At the indicated times, cells were fixed with 4% paraformaldehyde in 1× phosphate-buffered saline (1× PBS) for 10 min at room temperature (RT) and then permeabilized with ice-cold 0.1% Triton X-100 in PBS for 10 min at RT. Coverslips were washed in 1× PBS, blocked with 5% normal horse serum in 1× PBS at RT for 1 h and then rocked at 4°C with primary antibodies diluted in blocking buffer overnight. Primary antibodies used for IFA were rabbit anti-G3BP1 (1:300, Proteintech, #13057-2-AP), mouse anti-G3BP1 (1:300, Proteintech, #66486-1-Ig), rabbit anti-G3BP2 (1:200, Proteintech, #16276-1-AP), goat anti-TIA-1 (1:200, Santa Cruz Biotechnology, #1751), rabbit anti-Caprin-1 (1:50, Proteintech, #15112-1-AP), rabbit anti-USP10 (1:50, Cell Signaling Technology, #8501), rabbit anti-rpS6 (1:50, Cell Signaling Technology, #2217), rabbit anti-eIF3A (1:50, Cell Signaling Technology, #3314), rabbit anti-eIF4G (1:50, Cell Signaling Technology, #2498), rabbit anti-V5 (1:200, Cell Signaling Technology, #13202), mouse anti-V5 (1:200, Cell Signaling Technology, #80076), or mouse anti-dsRNA J2 (1:2,000, Nordic MUbio, #10010500). The coverslips were washed three times in PBS for 10 min, then incubated with either Alexa Fluor 555 donkey anti-mouse, Alexa Fluor 488 donkey anti-rabbit and/or Alexa Fluor 488 donkey anti-goat secondary antibodies (1:400, Thermo Fisher Scientific, #A31570, #A21206, #A11055, respectively) diluted in blocking buffer and Hoechst 33342 dye (0.08%, Invitrogen, #H3570) for 1 h at RT. The coverslips were washed with PBS and mounted onto glass slides with Prolong Gold Antifade reagent (Invitrogen, #P36930). Images were obtained using a 40× oil-immersion objective on a Zeiss Axio Observer Z1 widefield fluorescent microscope and were analyzed using Volocity software (Perkin Elmer).

### WB

Whole-cell lysates from mock-infected, transfected or SHFV-infected cells were harvested in 1× RIPA buffer (1× PBS, 1% Nonidet P-40, 0.5% sodium deoxycholate, and 0.1% SDS) containing Halt protease inhibitor cocktail (Thermo Fisher Scientific, #78430). Lysate proteins were separated on a 12% SDS-PAGE gel at 150 V for 1 h, and then transferred to a 0.2 µm nitrocellulose membrane using a Trans-Bolt Turbo Transfer System (Bio-Rad) following the manufacturer’s protocol. The membrane was incubated in blocking buffer (5% non-fat dry milk diluted in 1× Tris-buffered saline [TBS], pH 8.0, containing 0.1% Tween 20) at RT for 1 h, and then cut into strips and incubated with primary antibodies overnight at 4°C while rocking. The antibodies used were rabbit anti-PKR (1:400, Cell Signaling Technology, #12297), rabbit anti-p-PKR (1:500, Invitrogen, #MA5-38282), mouse anti-eIF2α (1:500, Abcam, #5369), rabbit anti-p-eIF2α (1:500, Abcam, #32157), mouse anti-G3BP1 (1:1,000, Proteintech, #66486-1-Ig), rabbit anti-G3BP1 (1:1,000, Thermo Fisher Scientific, #MA557405), rabbit anti-G3BP1 (1:1,000, Bethyl Laboratories, #A302-034A), rabbit anti-G3BP2 (1:1,000, Bethyl Laboratories, #A302-040A), mouse anti-V5 (1:500, Cell Signaling Technology, #80076), rabbit anti-nsp1α peptide (GDLTRPEETPLPGGC) (1:500) rabbit anti-nsp1β peptide (FAQKVITAFPEGVLC) (1:2,000), rabbit anti-nsp1γ peptide (FPPLSRKSEAQRAIL) (1:500), or mouse anti-beta actin (1:10,000, Abcam, #6276). The nsp1 antibodies are monospecific, polyclonal antibodies made by Abgent. The membrane strips were washed three times for 15 min with 1× TBST (1× TBS, pH 7.6 + 0.1% Tween) and then incubated with a secondary antibody (horseradish peroxidase-conjugated anti-rabbit or anti-mouse antibody) (1:2,000, Cell Signaling Technology, #7079, #7076, respectively) for 1 h at RT. The membrane strips were then washed two times in 1× TBST and once in 1× TBS for 15 min each and then developed with a Pierce ECL western blotting substrate (Thermo Fisher Scientific, #32106) following the manufacturer’s protocol.

### Co-immunoprecipitation assay

MA104 cells, seeded at 2 × 10^6^ cells/10 cm dish, were either mock-infected, infected, transfected, or infected and transfected. Whole-cell lysates from each plate were harvested in 500 µL of IP lysis buffer (25 mM Tris-HCl [pH 7.4], 150 mM NaCl, 1 mM EDTA, 1% NP-40, and 5% glycerol) containing Halt protease inhibitor cocktail (Thermo Fisher Scientific, #78430). Pre-cleared lysates (50 µL) were stored in −20°C for later use as the input controls for the WB analysis. The remaining lysates were separated into two aliquots. One aliquot was incubated with 1 µg of control rabbit anti-IgG antibody (Proteintech, #30000-0-AP) and the other was incubated with 1 µg of rabbit anti-G3BP1 antibody (Proteintech, #13057-2-AP). After 1 h with rotation at 4°C, Protein A magnetic beads (Cell Signaling Technology, #73778) were added to each sample and incubated overnight at 4°C with rotation. The beads were concentrated magnetically, washed three times in lysis buffer, and then incubated in 50 µL of 1× SDS sample loading buffer (10% SDS, 12.5% glycerol, 0.25 M Tris-HCl [pH 6.8], 0.25% bromophenol blue, and 2.5% beta-mercaptoethanol) for WB analysis or in 50 µL of 1× NuPAGE LDS sample loading buffer (Thermo Fisher Scientific, #NP0007) for LC-MS/MS sample preparation. Control input lysates were also incubated with SDS or LDS sample loading buffer. All samples were eluted by boiling for 10 min. The IP samples were separated from the magnetic beads, and the eluents were stored at −20°C until used for WB or LC-MS/MS analysis.

For immunoprecipitation of viral proteins with a V5 tag, the whole-cell lysates were harvested as described above and then incubated with 20 µL of V5-Trap Magnetic agarose bead slurry (Proteintech, #v5tma) at 4°C for 2 h with rotation. The beads were then centrifuged, pelleted, washed, and eluted in 1× sample loading buffer as described above.

### LC-MS/MS analysis

G3BP1 IP samples from mock or infected MA104 cells were prepared as described above. The precipitated proteins were run into a 12% NUPAGE bis-tris gel (Thermo Fisher Scientific, #NP0341BOX) using 1× MES SDS running buffer (Thermo Fisher Scientific, #NP0002) for a short distance. Precision Plus protein standards (Bio-Rad, #1610373) were run on a separate lane. The proteins were stained using a colloidal blue staining kit (Thermo Fisher Scientific, #LC6025) according to the manufacturer’s standard protocol. The entire stained gel region was excised as a single band from individual lanes, then reduced with Tris(2-carboxyethyl)phosphine, alkylated with iodoacetamide, and digested with trypsin (Promega, #V5111). Tryptic digests were analyzed using a 90 min LC gradient on a Thermo Q Exactive HF mass spectrometer (Thermo Fisher Scientific). LC-MS/MS data were searched with full tryptic specificity against the UniProt *Chlorocebus* proteome database (24 December 2022), SHFV protein sequences, and a common contaminant database using MaxQuant 1.6.3.3 (75). Protein and peptide lists were generated with a false discovery rate set at 1%. Statistical analyses were performed on protein LFQ Intensity values using LFQ-Analyst (76), and *P* values were adjusted to account for multiple testing using Benjamini-Hochberg FDR correction.

### *In silico* protein modeling

The protein sequences of the *Chlorocebus sabaeus* G3BP1 NTF2-L domain, SHFV nsp2, SHFV nsp3, SHFV N protein, SHEV N protein, and Mayaro virus nsp3 coding regions were used to generate predicted protein structures and interaction models using the AlphaFold 3 online server (https://golgi.sandbox.google.com). Analysis of the predicted interaction models and the crystal structure of the G3BP1-NTF2-L domain in complex with the SARS CoV2 N peptide ITFGGPS (PDB:7SUO) was carried out with the PyMOL Molecular Graphics System (v3.1.3.1, Schrödinger, LLC).

### GenBank accession numbers

The GenBank accession numbers for for G3BP1 and viral protein sequences used in alignments and *in silico* protein modeling are as follows: SHFV (NC_003092.2), PBJV (NC_027124.1), KKCBV-1 (KT447550), MYBV-1 (NC_025112.1), SWBV-1 (NC_025113.1), KRTGV-1 (JX473849.1), SHEV (KM677927.1), DeMAV (NC_026509.1), FSVV (KR862307.1), KRCV-2 (NC_034455.1), KRCV-1 (KX656702.1), ZMbV-1 (KT166441), SARS CoV2 (NC_045512.2), APRAV (NC_026439.1), RtEiAV (KY369968), MAYV (KT754168.1), and G3BP1 (XP_008013253.2).

## Data Availability

All data supporting the results of this study are available within the article and supplemental material. The LC-MS/MS proteomics data have been deposited into the MassIVE and ProteomeXchange data repositories with the accession numbers MSV000098034 and PXD064427, respectively.

## SUPPLEMENTAL MATERIAL

Table S1 jvi.00794-25-s0001.xlsx LC-MS/MS data for peptide analysis.

ASM does not own the copyrights to Supplemental Material that may be linked to, or accessed through, an article. The authors have granted ASM a non-exclusive, world-wide license to publish the Supplemental Material files. Please contact the corresponding author directly for reuse.
